# Study on the Antifungal Activity and Molecular Docking of Polyester Metabolites from *Talaromyces striatoconidius*

**DOI:** 10.3390/biology15120920

**Published:** 2026-06-12

**Authors:** Yanyan Chen, Mingjie Zhang, Siqin Li, Jiekang Xiao, Zheng Ma, Jiawen Sun, Xiachang Wang, Junwei Sun, Yongyong Zhang

**Affiliations:** 1 Department of Pharmacy, College of Life Sciences, China Jiliang University, Hangzhou 310018, China; p24091055007@cjlu.edu.cn (Y.C.); 2300903101@cjlu.edu.cn (M.Z.); 2300903131@cjlu.edu.cn (S.L.); 17870235892@163.com (J.X.); 2 Zhejiang Provincial Key Laboratory of Biometrology and Inspection & Quarantine, College of Life Sciences, China Jiliang University, Hangzhou 310018, China; mazheng520@163.com; 3 Jiangsu Key Laboratory for Functional Substances of Chinese Medicine, Nanjing University of Chinese Medicine, Nanjing 210023, China; 20230867@njucm.edu.cn (J.S.); xiachangwang@njucm.edu.cn (X.W.); 4 College of Modern Science and Technology, China Jiliang University, Yiwu 322002, China; 5 Key Laboratory of Marine Food Quality and Hazard Controlling Technology of Zhejiang Province, College of Life Sciences, China Jiliang University, Hangzhou 310018, China

**Keywords:** *Talaromyces striatoconidius*, polyesters, antifungal activity, *Fusarium oxysporum* f. sp. *cubense*, molecular docking, natural products

## Abstract

Plant pathogenic fungi seriously threaten global crop production, and endophytic fungi are promising reservoirs of bioactive antifungal metabolites. Three undescribed polyester derivatives, talapolyesters I–K (**1**–**3**), along with thirteen known compounds including 15G256ω (**4**), 15G256ι (**5**), 15G256α (**6**), talapolyester E (**7**), 15G256α-1 (**8**), 15G256α-2 (**9**), 15G256α-2-me (**10**), 15G256ν (**11**), ES-242-3 (**12**), dongtinganthracene A (**13**), penicillide (**14**), 3-methyl-6-hydroxy-8-methoxy-3,4-dihydroisocoumarin (**15**), and (*R*)-6-hydroxymellein (**16**), were isolated from the endophytic fungus *Talaromyces striatoconidius* WI-F2. Their chemical structures were elucidated comprehensively using NMR and MS spectroscopic analyses, combined with alkaline hydrolysis. Compounds **4**–**8** exhibited in vitro promising antifungal activity against *Fusarium oxysporum* f. sp. *cubense*, with half-maximal inhibitory concentration (IC_50_) values of 9.72, 21.07, 7.89, 8.91 and 9.65 μg/mL respectively, all markedly lower than that of ketoconazole (46.23 μg/mL). Molecular docking simulations further validated the observed antifungal activity, with binding energies ranging from −9.01 to −12.25 kcal/mol, indicative of stronger binding affinity compared with benzamidine (−6.80 kcal/mol). This study offers new clues for research on antifungal agents.

## Simple Summary

Fungal diseases are a major threat to crop production worldwide, causing serious yield losses. New antifungal compounds from natural sources are therefore urgently needed. In this study, we isolated several natural products from a fungus that lives inside plant roots. We found five of these compounds were highly effective at stopping the growth of the harmful *Fusarium oxysporum* f. sp. *cubense*, which causes devastating plant diseases. Our computer simulations showed these compounds bind tightly to a key protein in the fungus, blocking its function. These findings provide promising new leads for developing safer and more effective antifungal treatments to protect crops.

## 1. Introduction

Natural polyesters are recognized as one of the six major classes of natural polymers, which also include proteins, polysaccharides, polynucleotides, polyisoprenes and lignin. Structurally, natural polyesters are condensation polymers composed of monomeric units covalently connected via repetitive ester bonds along the molecular backbone [1,2]. They are ubiquitously distributed in the secondary metabolites of various fungal species and exhibit a broad spectrum of biological activities, such as antifungal [3,4,5], cytotoxic [6,7], antimalarial [8,9], and antibacterial [10,11,12] effects. As an important fungal resource, the genus *Talaromyces* has attracted considerable attention, from which a large number of polyester derivatives, including talapolyesters A–H, have been isolated and identified [13,14,15,16].

Endophytic fungi undergo long-term co-evolution with their host plants and thereby produce structurally unique and chemically diversified secondary metabolites, representing a largely untapped source for mining novel bioactive lead scaffolds. Among these endosymbionts, the ascomycete *Talaromyces striatoconidius*, a typical member of the well-studied *Talaromyces* genus, stands out as a promising producer of structurally varied polyketide and polyester metabolites, thus representing a largely untapped source for mining novel bioactive lead scaffolds [17]. In continuation of our ongoing program aimed at exploring bioactive constituents from fungal resources, an endophytic strain WI-F2 was isolated from the medicinal herb *Wikstroemia indica* and taxonomically identified as *T. striatoconidius* WI-F2 by molecular phylogenetic analysis.

To further explore its practical antifungal application potential, we selected the destructive plant pathogen *Fusarium oxysporum* as the screening target. *F. oxysporum*, a devastating soil-borne phytopathogenic fungus, triggers destructive vascular wilt diseases across dozens of economically vital crops worldwide [18]. Its forma specialis *cubense* is the causative agent of Panama disease in banana, leading to massive global crop yield losses annually [19,20]. Given the urgent demand for new antifungal leads against this destructive pathogen, we prioritized *F. oxysporum* f. sp. *cubense* as the test strain for our in vitro antifungal bioassay.

Although many polyester natural products have been characterized from various *Talaromyces* strains in previous chemical investigations, little information is available concerning secondary metabolites derived from the endophytic fungus *T. striatoconidius*, especially their inhibitory potential toward the destructive plant pathogen *F. oxysporum* f. sp. *cubense*. Accordingly, the present work was initiated to explore its bioactive constituents via chromatographic purification, structural elucidation and in vitro antifungal evaluation, supplemented with molecular docking to clarify binding interaction modes.

## 2. Materials and Methods

### 2.1. General Experimental Procedures and Equipment

Optical rotations were measured on an Anton Paar MCP 150 Digital Polarimeter (Anton Paar GmbH, Graz, Austria). UV spectra were recorded in methanol on a Shimadzu UV-2401A spectrophotometer (Shimadzu Corporation, Kyoto, Japan). Liquid chromatography-mass spectrometry (LC-MS) analyses were performed with an Agilent 6120 Quadrupole MSD mass spectrometer (Agilent Technologies Inc., Santa Clara, CA, USA), and HRESIMS measurements were obtained with a Thermo QE-HF-X high-resolution mass spectrometer (Thermo Fisher Scientific Inc., Waltham, MA, USA). One- and two-dimensional NMR spectra were measured on Bruker AVANCE NEO 400 MHz and AVANCE III 500 MHz NMR spectrometers (Bruker BioSpin AG, Fällanden, Switzerland). Semi-preparative HPLC was conducted on a Shimadzu LC-6AD system equipped with a photodiode array (PDA) detector (Shimadzu Corporation, Kyoto, Japan), using a PotenSil C18 column (20 × 250 mm, 5 μm). Column chromatography (CC) was carried out on AmberLite XAD-16N macroporous resin (Sigma-Aldrich Co., St. Louis, MO, USA), Daisogel ODS silica gel (75 μm) (DAISO Co., Ltd., Osaka, Japan) and Sephadex LH-20 (25–100 μm) (GE Healthcare, Uppsala, Sweden, Pharmacia). All fractions were monitored by thin-layer chromatography (TLC). Spots were visualized under UV light at 254 nm, followed by spraying with sulfuric acid-anhydrous ethanol solution and subsequent heating. The tested strain *Fusarium oxysporum* f. sp. *cubense* (CGMCC 3.12196) was obtained from the China General Microbiological Culture Collection Center (CGMCC) (Institute of Microbiology, Chinese Academy of Sciences, Beijing, China).

### 2.2. Fungal Material

The fungal strain *T. striatoconidius* WI-F2 was isolated from the root of *Wikstroemia indica* collected in Zhejiang Province, China. The isolation procedure was consistent with that described in our previous study [21]. The ITS sequence of this strain was submitted to GenBank with accession code PZ413744, and the living culture specimen is conserved in the microbial collection housed at the School of Life Sciences, China Jiliang University.

### 2.3. Fermentation, Extraction and Isolation

The fungal strains were cultured on potato dextrose agar (PDA) at 28 °C for 5 days. Mycelia and spores were scraped from each of the 15 PDA plates with 5 mL of sterile water. All harvested suspensions were pooled, and the total volume was brought to 600 mL for preparation of the fungal inoculum. An aliquot (2 mL) of the resulting suspension was inoculated into each of 300 Erlenmeyer flasks (250 mL). The culture formulation per flask consisted of 16 g oats, 0.5 g pancreatic tryptone supplemented with 60 mL purified water, and the culture medium was sterilized by autoclaving prior to use. After incubation at 28 °C for 21 days, the fermented oat substrate was extracted three times with ethyl acetate. The organic extracts were combined and concentrated under reduced pressure with rotary evaporation in vacuo to concentrate into a crude extract residue. The resultant crude residue was resuspended in distilled water and sequentially partitioned with *n*-hexane, dichloromethane, and ethyl acetate. Each organic fraction was concentrated under reduced pressure to give three crude extracts. Guided by TLC and HPLC analysis, the dichloromethane and ethyl acetate fractions were combined and further partitioned between methanol and water to yield the methanol-soluble fraction and aqueous fraction, respectively.

The aqueous extract was loaded onto an XAD-16N macroporous resin column and subjected to step-gradient elution by MeOH/H_2_O (0% → 100% MeOH in six steps), yielding five main fractions (Fr. A1–A5). Fr. A2 was further fractionated by Sephadex LH-20 column chromatography using isocratic methanol elution, yielding four primary subfractions (Fr. B1–B4). Fractions B2 and B3 were combined and purified by semi-preparative HPLC using a linear gradient of MeCN–H_2_O (20:80 to 30:70, *v*/*v*) at a flow rate of 6.0 mL/min, with UV detection at 254 nm, to afford compound **15** (*t*_R_ = 40 min, 69.0 mg).

The methanol extract was also fractionated over a macroporous resin column and eluted with a serial MeOH–H_2_O gradient (0% to 100% MeOH) for step elution, affording five principal fractions (Fr. C1–C5). Fr. C2 yielded an abundant pale-yellow precipitate, which was recrystallized from MeOH to give compound **12** (147.0 mg). Fr. C3 was then subjected to ODS column chromatography using a stepwise MeOH–H_2_O gradient (30%, 35%, 40%, 45%, 50%, 55%, 60%, 70%, 80%, and 100% MeOH) to furnish eleven subfractions (Fr. D1–D11). Fr. D3 produced a pale precipitate, which was recrystallized from MeOH to afford compound **16** (176.0 mg) as colorless needle crystals. Fr. D5 was subjected to semi-preparative RP-HPLC with a linear mobile phase gradient of MeCN–H_2_O (65:35 to 70:30, *v*/*v*) to yield compound **4** (*t*_R_ = 42 min, 60.0 mg). Similarly, Fr. D7 was purified using a MeCN–H_2_O gradient (50:50 to 62:38, *v*/*v*) to afford compounds **9** (*t*_R_ = 16 min, 51.6 mg), **2** (*t*_R_ = 24 min, 31.7 mg), **1** (*t*_R_ = 25 min, 58.4 mg), **10** (*t*_R_ = 31 min, 9.0 mg), **6** (*t*_R_ = 37 min, 28.1 mg), **8** (*t*_R_ = 45 min, 18.3 mg) and **3** (*t*_R_ = 49 min, 38.9 mg). Fr. D8, using a linear gradient of MeCN–H_2_O (50:50 to 78:22, *v*/*v*), produced compounds **7** (*t*_R_ = 42 min, 33.1 mg) and **5** (*t*_R_ = 43 min, 28.9 mg). Fr. D9 further refined via semi-preparative HPLC eluting with MeCN–H_2_O gradient (57:43 to 62:38, *v*/*v*), yielding compound **14** (*t*_R_ = 50 min, 8.9 mg). Fr. D10 was purified under a gradient of MeCN–H_2_O (48:52 to 70:30, *v*/*v*) to afford compound **11** (*t*_R_ = 16 min, 23.9 mg). Fr. D11, using a MeCN–H_2_O gradient of 47:53 to 70:30 (*v*/*v*), gave compound **13** (*t*_R_ = 40 min, 30.0 mg).

Talapolyester I (**1**): colorless powder; [*α*]25D –32.0 (*c* 0.1, MeOH); UV (MeOH) λ_max_ (log *ε*) 216 (4.23), 264 (3.98), 302 (3.67) nm; LC-MS *m*/*z* 721.2 [M−H]^−^; HRESIMS *m*/*z* 721.2204 [M−H]^−^ (calcd. for C_34_H_41_O_17_, 721.2344); ^1^H NMR (CD_3_OD, 400 MHz); and ^13^C NMR (CD_3_OD, 100 MHz) in Table 1.

Talapolyester J (**2**): colorless powder; [*α*]25D –41.0 (*c* 0.1, MeOH); UV (MeOH) λ_max_ (log *ε*) 216 (4.57), 266 (4.31), 305 (3.94) nm; LC-MS *m*/*z* 721.4 [M−H]^−^; HRESIMS *m*/*z* 721.2202 [M−H]^−^; (calcd. for C_34_H_41_O_17_, 721.2344); ^1^H NMR (CD_3_OD, 500 MHz); and ^13^C NMR (CD_3_OD, 125 MHz) in Table 1.

Talapolyester K (**3**): colorless powder; [*α*]25D –68.0 (*c* 0.1, MeOH); UV (MeOH) λ_max_ (log *ε*) 214 (4.55), 262 (4.29), 301 (3.93) nm; LC-MS *m*/*z* 735.2 [M−H]^−^; HRESIMS *m*/*z* 735.2366 [M−H]^−^; (calcd. for C_35_H_43_O_17_, 735.2500); ^1^H NMR (CD_3_OD, 400 MHz); and ^13^C NMR (CD_3_OD, 125 MHz) in Table 1.

### 2.4. Antifungal Activity Assays In Vitro

The in vitro antifungal activity assay protocol was performed following our previously established method [22]. For inoculum preparation, *Fusarium oxysporum* f. sp. *cubense* was cultured on PDA medium at 28 °C for 7 days, and the resulting conidia were harvested by flooding the plates with sterile 0.1% Tween 80 solution. The conidial suspension was then filtered through four layers of sterile gauze, and the concentration was adjusted to 1 × 10^6^ conidia/mL using a hemocytometer.

All compounds were dissolved in methanol to prepare stock solutions, which were then blended with sterile PDA medium preheated to 55 °C to prepare culture media containing final compound concentrations of 160, 80, 40, 20, and 10 μg/mL. The final concentration of methanol in all media, including the negative control, was kept at 0.5% (*v*/*v*) to eliminate any potential solvent effect. The media were subsequently poured into Petri dishes (60 mm in diameter). After solidification, 2 μL of the standardized fungal conidial suspension was inoculated onto the center of each plate. All plates were incubated at 28 °C for 7 days, or until the mycelia of the negative control group fully spread to the edge of the Petri dish.

The colony diameter of each plate was measured twice by the cross-crossing method, and the mean value was recorded. The mycelial growth inhibition rate (%*GI*) was calculated by the formula

%*GI* = [(*C* − *T*)/*C*] × 100,

in which *C* and *T* denote the average colony diameters of the negative control group and experimental treatment group, respectively. Methanol (0.5%, *v*/*v*) was used as the negative control, and ketoconazole was set as the positive control. All experiments were conducted in triplicate and the results are presented as mean ± standard deviation (SD).

The half-maximal inhibitory concentration (IC_50_) represents the compound concentration producing 50% suppression of fungal mycelial growth. Based on the obtained growth inhibition percentages at five gradient concentrations, IC_50_ values were calculated via probit regression analysis using SPSS 26.0 software, with final results expressed as mean ± standard deviation.

### 2.5. Molecular Docking Study

In silico docking calculations with AutoDock Tools v1.5.7 [23] were performed to explore the binding affinity and intermolecular interaction patterns between the isolated compounds and the target protein [24,25,26,27]. The crystal structure of trypsin-like serine protease from *F. oxysporum* (PDB ID: 1FN8) [28] was retrieved from the RCSB PDB database. This protease was selected as docking receptor owing to its indispensable function in pathogen infection and host tissue degradation of *F. oxysporum* f. sp. *cubense* [29,30,31], followed by protonation and energy minimization in PyMoL v4.6 (Schrödinger LLC, New York, NY, USA) to prepare the receptor. Meanwhile, the isolated compounds were constructed and optimized as ligands using ChemDraw v18.1 (Revvity Signals Software, Waltham, MA, USA). The ligand binding sites within the protein structure were identified using ProteinsPlus web server (https://proteins.plus/). Both the ligand molecules (including the positive control) and the receptor were subjected to molecular docking with default parameter settings. Subsequently, the docking results were analyzed and compared with those of the control group. Intermolecular interaction analyses were carried out via AutoDock Analyzer. Visualization of docking interactions was accomplished using PyMOL and LigPlot+ v2.3.1 [32]. The corresponding binding energy, hydrogen bonds with residues and hydrophobic contacts with residues were used to evaluate the conformational stability and binding potency of the complexes.

### 2.6. Alkaline Hydrolysis

Compounds **1**–**3** underwent alkaline hydrolysis based on the documented procedure [10] after minor revisions. Briefly, each sample (20.0 mg) was dissolved in 10.0 mL of 1 M aqueous NaOH solution and stirred at room temperature for 24 h. The reaction was terminated by acidification with 10.0 mL of 1 M HCl. The resulting mixture was concentrated under reduced pressure to yield an orange-red solid. The obtained solid was redissolved in anhydrous methanol, and insoluble sodium chloride was removed via filtration. The filtrate was concentrated in vacuo to afford an orange-red residue, which was subsequently dissolved in 50 mL of dry benzene. A catalytic amount of para-toluene sulfonic acid was added, and the mixture was heated under reflux for 30 min. After cooling to room temperature, the benzene phase was successively washed with saturated sodium bicarbonate solution and distilled water, then dried over anhydrous sodium sulfate. Following vacuum concentration, the crude product was purified by semi-preparative HPLC. The isolated components were unambiguously identified by comparison of their ^1^H NMR, MS and specific optical rotation data with those reported in the literature [10,33].

(−)-(*R*)-3-hydroxybutyric acid: colorless oil; [*α*]25D –31 (*c* 0.1, MeOH); ESIMS *m*/*z* 103.2 [M−H]^−^; ^1^H NMR (CD_3_OD, 400 MHz) *δ* 4.03 (1H, m), 2.23 (2H, m), 1.10 (3H, d, *J* = 5.6 Hz).

(−)-(*S*)-3,4-dihydroxybutyric acid-γ-lactone: colorless oil; [*α*]25D –66 (*c* 0.1, MeOH); ESIMS *m*/*z* 101.2 [M−H]^−^; ^1^H NMR (CD_3_OD, 400 MHz) *δ* 4.35 (1H, m), 4.30 (1H, dd, *J* = 10.6, 4.5 Hz), 4.11 (1H, dd, *J* = 10.5, 1.0 Hz), 2.79 (1H, dd, *J* = 17.8, 5.8 Hz), 2.39 (1H, dd, *J* = 17.8, 1.2 Hz).

(−)-(*R*)-6-hydroxymellein: colorless powder; [*α*]25D –56 (*c* 0.1, MeOH); ESIMS *m*/*z* 193.0 [M−H]^−^; ^1^H NMR (CD_3_OD, 500 MHz) *δ* 6.22 (1H, overlapped), 6.21 (1H, overlapped), 4.66 (1H, m), 2.91 (1H, dd, *J* = 16.4, 3.5 Hz), 2.83 (1H, dd, *J* = 16.4, 11.1 Hz), 1.27 (3H, d, *J* = 6.3 Hz).

## 3. Results

**Figure 1:**
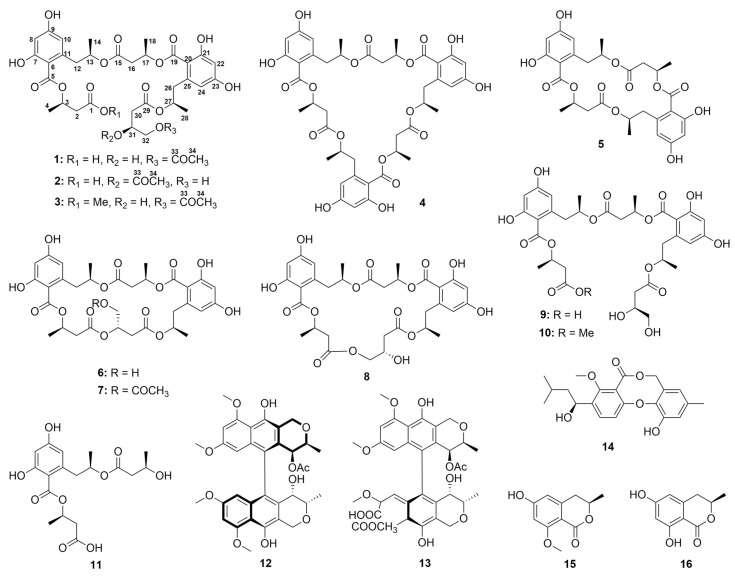
Structures of compounds **1**–**16**.

Strain WI-F2 was subjected to scale-up fermentation, which led to the isolation and structural elucidation of three undescribed polyester compounds, namely talapolyesters I–K (**1**–**3**), together with thirteen previously reported compounds (**4**–**16**) (Figure 1). Among them, compounds **4**–**8** displayed remarkable inhibitory effects against *F*. *oxysporum* f. sp. *cubense*. This finding is consistent with previous reported literature [5] that cyclic polyesters exert superior antifungal potency, whereas linear polyesters are generally inactive, which was further supported by molecular docking analysis. To the best of our knowledge, the present work represents the first report on the antifungal evaluation and molecular docking study of natural polyesters against *F. oxysporum* f. sp. *cubense*.

### 3.1. Structural Elucidation

**Figure 2:**
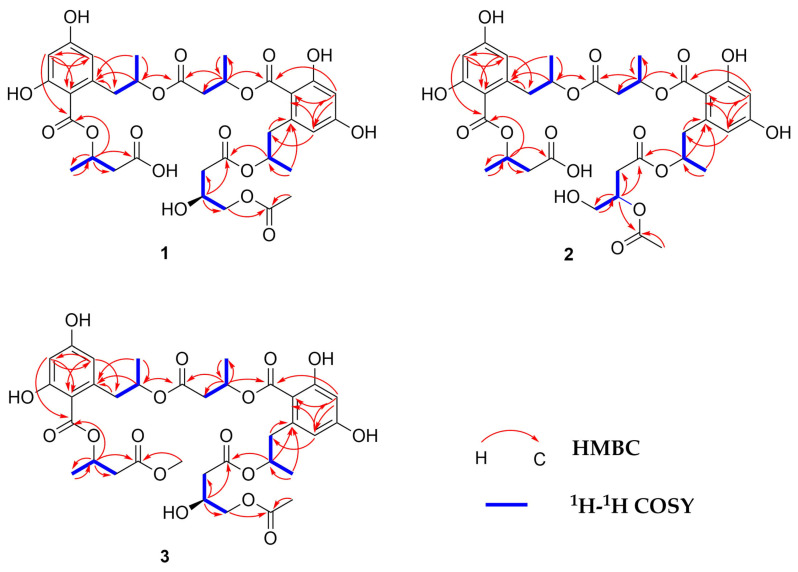
Key HMBC (red arrows) and ^1^H−^1^H COSY (bold blue lines) correlations of compounds **1**–**3**.

Compound **1** was obtained as a colorless powder. Its molecular formula was determined based on HRESIMS as C_34_H_42_O_17_ with 14 degrees of unsaturation. The ^1^H and ^13^C NMR data of 1 (Table 1) were closely identical to those of talapolyester E (**7**), whereas comparison of their molecular formulas revealed that **1** contained one additional oxygen atom and two more hydrogen atoms compared with **7**. Significant NMR differences between these two compounds were observed in the downfield shifts in C-1 (*δ*_C_ 173.9), C-30 (*δ*_C_ 40.0), and C-32 (*δ*_C_ 68.3), as well as in the upfield shift in C-31 (*δ*_C_ 67.0) of **1**, compared with those of **7** (C-1, *δ*_C_ 171.3; C-30, *δ*_C_ 36.7; C-32, *δ*_C_ 65.4; and C-31, *δ*_C_ 70.3). These data suggested that the ester linkage between C-1 and C-31 was cleaved upon the addition of one molecule of water, leading to the conversion of the cyclic structure into an acyclic form. Similarly, **1** also contained two units of 2,4-dihydroxy-6-(2-hydroxypropyl) benzoic acid, two units of 3-hydroxybutyric acid, and one unit of 4-acetoxy-3-hydroxybutyric acid. All the units were linked via ester bonds as supported by HMBC correlations from H-3 to C-5, H-13 to C-15, H-17 to C-19, and H-27 to C-29 (Figure 2).

Given that 2,4-dihydroxy-6-(2-hydroxypropyl) benzoic acid is the linear, ring-opened analogue of 6-hydroxymellein, and 4-acetoxy-3-hydroxybutyric acid is the 4-O-acetylated derivative of 3,4-dihydroxybutyric acid [5,13], the absolute configuration of each residue in **1** were unambiguously established by comparing the specific optical rotations of its alkaline hydrolysates with literature data. The hydrolysates were identified as (−)-(*R*)-6-hydroxymellein ([α]25D −56, *c* 0.1, MeOH) [5,10], (−)-(*R*)-3-hydroxybutyric acid ([α]25D −31, *c* 0.1, MeOH) [10] and (−)-(*S*)-3,4-dihydroxybutyric acid-γ-lactone ([α]25D −66, *c* 0.1, MeOH) [3,13]. Accordingly, the absolute configuration of each structural subunit was rationally inferred. Consequently, the absolute stereochemistry of compound **1** was elucidated and named as talapolyester I.

Compound **2** was yielded as a colorless powder. Its molecular formula was established as C_34_H_42_O_17_ by HRESIMS with 14 degrees of unsaturation, which was identical to that of compound **1**. NMR spectroscopic data showed that compound **2** was closely related to compound **1** (Table 1). Relative to compound **1**, compound **2** exhibited a downfield shift for C-31 and upfield shifts for C-30 and C-32, indicating that the acetyl group was located at C-31 in **2** rather than at C-32 in **1**. This assignment was confirmed by HMBC correlations: a correlation from H-31 to C-33 was observed in **2** (Figure 2), whereas the correlation from H-32 to C-33 was present in **1**. Similarly, the absolute configuration of **2** was determined by alkaline hydrolysis. Therefore, the structure of **2** was deduced and named as talapolyester J.

Compound **3** was assigned the molecular formula of C_35_H_44_O_17_, corresponding to 14 degrees of unsaturation, on the basis of HRESIMS analysis. Its ^1^H and ^13^C NMR spectroscopic data were highly consistent with those of compound **1**, with the only discrepancy being the appearance of a methoxy signal at *δ*_H_ 3.66 (*δ*_C_ 52.3). HMBC experiment verified the attachment of this methoxy group to C-1 (*δ*_C_ 172.4) (Figure 2), thus identifying **3** as a methoxylated derivative of **1**. By reference to the alkaline hydrolysis products and their optical rotation values of **1**, the absolute configuration of **3** was fully characterized and named as talapolyester K.

Furthermore, thirteen known compounds were identified by comparing their NMR, mass spectral data and specific optical rotations with published literature data. These compounds were elucidated as 15G256ω (**4**) [5], 15G256ι (**5**) [5], 15G256α (**6**) [5], talapolyester E (**7**) [13], 15G256α-1 (**8**) [5], 15G256α-2 (**9**) [5], 15G256α-2-me (**10**) [5], 15G256ν (**11**) [5], ES-242-3 (**12**) [34], dongtinganthracene A (**13**) [35], penicillide (**14**) [36], 3-methyl-6-hydroxy-8-methoxy-3,4-dihydroisocoumarin (**15**) [37], and (*R*)-6-hydroxymellein (**16**) [13].

### 3.2. Antifungal Activity In Vitro

**Figure 3:**
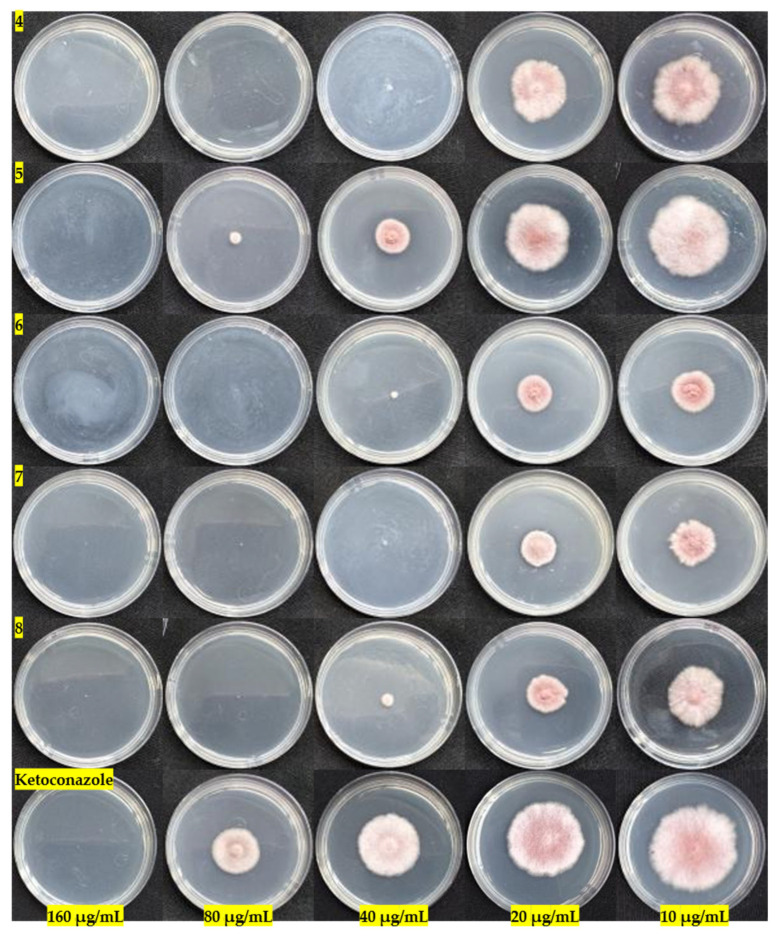
In vitro antifungal activity of compounds **4**–**8** against *F. oxysporum* f. sp. *cubense*.

In vitro antifungal potency of all isolated metabolites was assessed against *F. oxysporum* f. sp. *cubense* using the mycelial growth inhibition assay. Of the tested compounds, only compounds **4**–**8** exhibited measurable antifungal activity, aligning with previously published observations that cyclic polyesters confer antifungal activity, in contrast to their inactive acyclic analogs. As illustrated in Table 2 and Figure 3, compounds **4**, **6**, **7** and **8** displayed the most potent antifungal efficacy, with IC_50_ values all below 10 μg/mL (9.72, 7.89, 8.91 and 9.65 μg/mL, respectively), while compound **5** had an IC_50_ value of 21.07 μg/mL. Markedly, the IC_50_ values of all active compounds were significantly lower than that of the positive control ketoconazole (IC_50_ = 46.23 μg/mL).

### 3.3. Molecular Docking Study for Antifungal Activity

**Figure 4:**
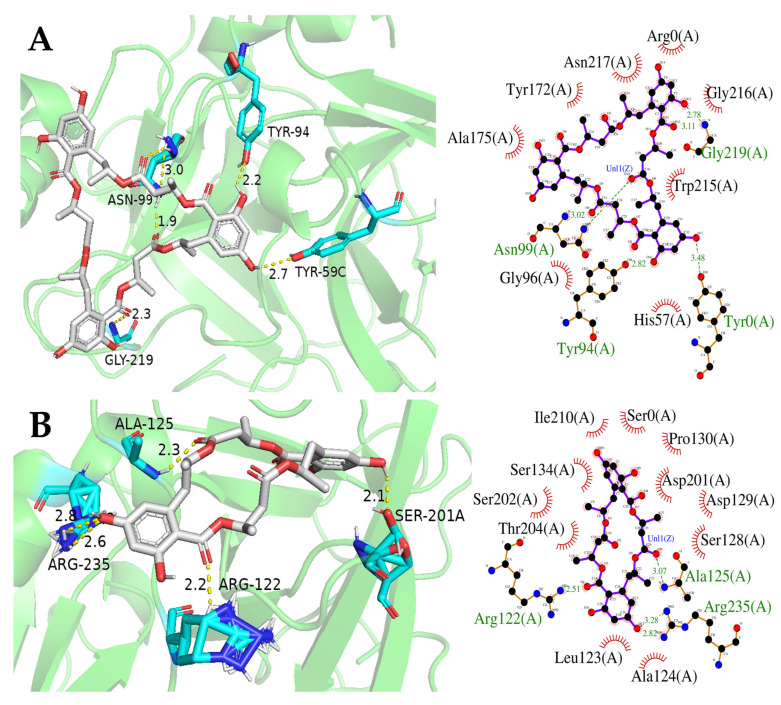

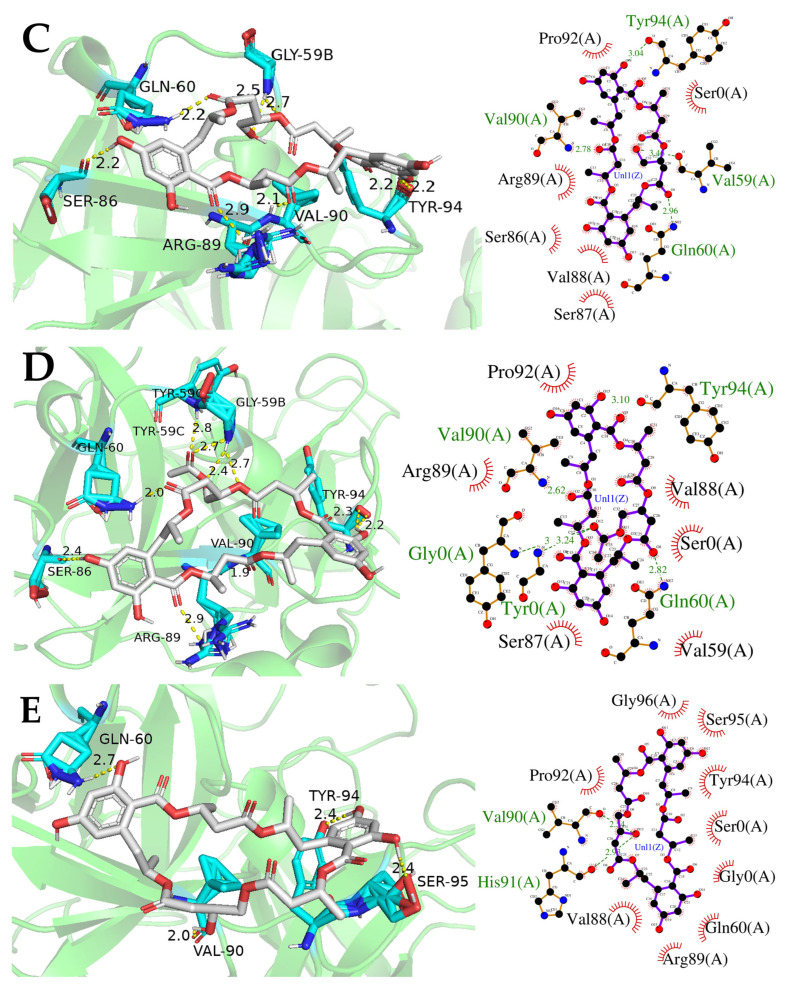

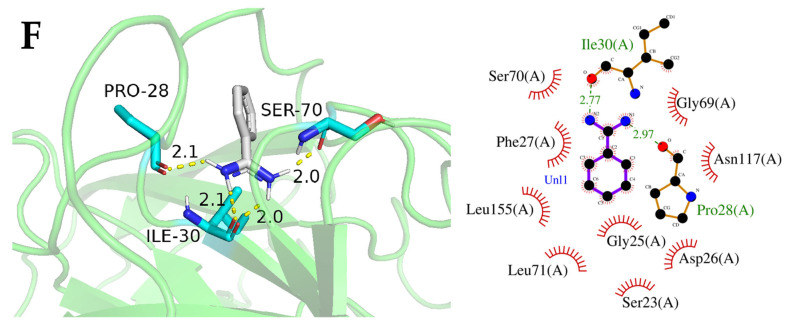
Three-dimensional (3D) and two-dimensional (2D) docking interaction diagrams of compounds **4**–**8** and benzamidine within the active site of the target protein (PDB ID: 1FN8) from *Fusarium oxysporum*. (**A**) Compound **4**; (**B**) Compound **5**; (**C**) Compound **6**; (**D**) Compound **7**; (**E**) Compound **8**; (**F**) Benzamidine. Yellow dashed lines indicate hydrogen bonds, and red arcs represent hydrophobic interactions between the ligands and protein residues.

All docking simulations were performed on the protease from *F. oxysporum*, the pivotal pathogenic enzyme chosen as target in the present work. As a crucial parameter, docking-derived binding free energy (Δ*G*) is widely used to judge ligand-receptor binding capacity. Negative Δ*G* values indicate energy release during the binding process, suggesting that the formed ligand–protein complex possesses higher thermodynamic stability than free individual molecules. All Δ*G* values of the isolated compounds are summarized in Table 3, and the optimal two-dimensional and three-dimensional binding conformations of ligands are displayed in Figure 4. Compounds **4**–**8** exhibited prominent binding affinity toward *F. oxysporum*, with corresponding binding free energies of −9.53, −9.01, −9.94, −12.25 and −9.41 kcal/mol, all of which were lower than that of benzamidine (−6.80 kcal/mol). Collectively, these molecular docking findings were consistent with in vitro antifungal results, suggesting that the five compounds exert stronger biological activity than benzamidine.

## 4. Discussion

According to the classic screening assay described by Kirsch et al. [38], polyesters, particularly cyclic polyesters, are well documented to exert antifungal effects by targeting fungal cell walls. These compounds specifically inhibit the polymerization of chitin and β-glucan, rather than directly killing fungal protoplasts [4,5,39]. In the present work, we conducted in vitro antifungal assays to verify this property. All five isolated cyclic polyesters (compounds **4**–**8**) showed potent inhibitory activity against *Fusarium oxysporum* f. sp. *cubense*, which is consistent with previous findings on cell-wall-active polyesters.

Based on existing literature and our phenotypic observations, these polyesters likely act via two complementary antifungal mechanisms. On the one hand, they interfere with chitin and β-glucan biosynthesis to disrupt the cell wall integrity of the pathogen. On the other hand, they may also inhibit key pathogenic enzymes, thereby limiting fungal infection and growth. To explore the second mechanism at the molecular level, we performed molecular docking simulations against trypsin-like serine protease from *F. oxysporum* (PDB ID: 1FN8). This enzyme plays essential roles in fungal nutrient acquisition and host tissue degradation, and thus represents a promising antifungal target. Its use in docking studies of natural products against fungal proteases has been validated in previous work [29,30,31]. It should be noted that the selection of this structure was based on its high sequence homology to the target protease and its well-defined three-dimensional conformation, making it a suitable template for molecular modeling. While the docking results provided correlative evidence supporting protease inhibition as a contributing mechanism, definitive confirmation awaits future in vitro enzyme inhibition assays.

According to the empirical criterion widely used in virtual screening, compounds with Δ*G* ≤ −7 kcal/mol are generally considered to have potential bioactivity; those with Δ*G* ranging from −7 to −8.9 kcal/mol exhibit moderate to strong affinity; and those with Δ*G* ≤ −9 kcal/mol are regarded as high-affinity binders. This classification is derived from the thermodynamic relationship between binding free energy and inhibition constant, and has been validated by long-term practical applications in the field [40,41]. As shown in Table 3, the Δ*G* values of compounds **4**–**8** ranged from −9.01 to −12.25 kcal/mol, indicating that these cyclic polyesters acted as high-affinity binders.

As illustrated in Figure 4, compounds **4**–**8** formed extensive hydrogen bonds and hydrophobic interactions with key amino acid residues within the active pocket. The superior intermolecular interactions of the tested polyesters well supported their prominent in vitro antifungal performance. All active compounds among compounds **4**–**8** readily occupied the catalytic pocket of the protease, where multiple hydrogen bonds with catalytic residues and widespread hydrophobic contacts with surrounding nonpolar residues jointly stabilized the ligand–protein complexes. Such binding effectively blocked substrate access to the active site and suppressed the protease function associated with host invasion and nutrient metabolism of *F. oxysporum*. The good correlation between Δ*G* values and experimental IC_50_ further demonstrated that inhibition of this serine protease is one major antifungal mechanism of the tested polyesters.

Compounds **1**–**3** are open-chain congeners of the cyclic talapolyesters. They lack the macrocyclic ester bond, showing increased molecular flexibility, and differ in site-specific acetylation (C-32 in **1**/**3**, C-31 in **2**) and terminal methylation (in **3**). These linear skeletons likely represent biosynthetic shunt metabolites of the pathway, reflecting incomplete cyclization and the action of flexible tailoring enzymes. However, both in vitro antifungal assays and in silico molecular docking revealed that these acyclic compounds showed no obvious bioactivity against *Fusarium oxysporum*. From the perspective of structure–activity relationships (SAR), cyclic polyesters exhibit superior binding affinity compared with acyclic analogues, mainly due to conformational restriction. The cyclic skeleton locks the molecule into a conformation that well matches the active pocket of the target protease, reducing the energy cost for conformational rearrangement during binding. Meanwhile, the fixed spatial arrangement of hydroxyl and carbonyl groups facilitates the formation of stable hydrogen bond networks and stronger hydrophobic interactions. In contrast, highly flexible acyclic polyesters undergo random torsion in solution, which weakens intermolecular forces and results in poorer binding capacity and lower antifungal activity. These results enriched the SAR of polyesters and demonstrated that the macrocyclic skeleton is indispensable for potent antifungal activity. Among compounds **4**–**8**, compounds **6**–**8** all contain two units of 2,4-dihydroxy-6-(2-hydroxypropyl)benzoic acid, two units of 3-hydroxybutyric acid, and one unit of 3,4-dihydroxybutyric acid. Notably, compound **7** bears an additional acetyl group and displayed the lowest Δ*G*, suggesting that the acetyl moiety is a critical structural group enhancing binding potency. Although compounds **6** and **8** share the same building blocks, their differing cyclization positions lead to distinct molecular conformations, which further affect their intermolecular interactions and result in different Δ*G*. Compound **4** possesses three units of 2,4-dihydroxy-6-(2-hydroxypropyl)benzoic acid and three units of 3-hydroxybutyric acid, whereas compound **5** contains only two of each. Consequently, compound **4** provided more interaction sites with the target protein, which is consistent with its stronger Δ*G* compared to compound **5**. Nevertheless, the Δ*G* of compound **4** is comparable to those of compounds **6** and **8**, implying that all three types of structural fragments contribute cooperatively to the binding affinity.

Overall, combining in vitro antifungal data and molecular docking results, these isolated cyclic polyesters presumably exert dual antifungal modes of action. On one hand, they interfere with chitin and β-glucan biosynthesis to disrupt the cell wall integrity of *F. oxysporum* f. sp. *cubense*. On the other hand, they bind to the protease and inhibit the enzyme activity critical for fungal pathogenicity.

## 5. Conclusions

In summary, three new polyester derivatives (compounds **1**–**3**) and thirteen known compounds were obtained from the metabolites of the endophytic fungus *T*. *striatoconidius* WI-F2. All isolated compounds were evaluated for their in vitro antifungal activity. While the new isolates displayed no remarkable antifungal effects, known cyclic polyesters **4**–**8** exhibited prominent antifungal potency, which was further verified by molecular docking simulations. Docking analyses indicated that these cyclic polyesters are promising candidates for the development of antifungal agents.

## Figures and Tables

**Table 1:** ^1^H and ^13^C NMR data for compounds **1**–**3** in CD_3_OD.

No.	1 ^a^		2 ^b^		3 ^c^	
	*δ*_C_, Type	*δ*_H_, Mult. (*J* in Hz)	*δ*_C_, Type	*δ*_H_, Mult. (*J* in Hz)	*δ*_C_, Type	*δ*_H_, Mult. (*J* in Hz)
1	173.9, C		174.1 ^d^, C		172.4, C	
2	41.4, CH_2_	2.69 (overlapped); 2.67 (overlapped)	41.8, CH_2_	2.73 (overlapped); 2.70 (overlapped)	41.2, CH_2_	2.78 (overlapped); 2.75 (overlapped)
3	70.5, CH	5.52, m	70.7, CH	5.56, m	70.2, CH	5.55, m
4	20.1, CH_3_	1.38, d (6.3)	20.2, CH_3_	1.42, d (6.3)	20.1, CH_3_	1.40, d (6.4)
5	171.6, C		171.7, C		171.6, C	
6	105.7, C		105.7, C		105.7, C	
7	166.2, C		166.2, C		166.3, C	
8	102.8, CH	6.15 (overlapped)	102.9, CH	6.20 (overlapped)	102.8, CH	6.19 (overlapped)
9	163.4, C		163.5, C		163.6, C	
10	113.8, CH	6.18, d (2.5)	113.9, CH	6.22, d (2.5)	113.9, CH	6.22, d (2.5)
11	143.8, C		143.8, C		143.7, C	
12	43.4, CH_2_	2.82, dd (13.5, 9.5); 2.74, dd (13.5, 4.7)	43.5, CH_2_	3.28, dd (13.5, 3.8); 2.86, dd (13.5, 9.6)	43.3, CH_2_	2.91, dd (13.5, 9.2); 2.77 (overlapped)
13	73.1, CH	5.16, m	73.3, CH	5.19, m	73.1, CH	5.15, m
14	20.5, CH_3_	1.20, d (6.3)	20.6, CH_3_	1.22, d (6.2)	20.5, CH_3_	1.25, d (6.3)
15	171.1, C		171.2, C		171.1, C	
16	41.7, CH_2_	2.62, m; 2.60, m	41.8, CH_2_	2.65 (overlapped)	41.8, CH_2_	2.66 (overlapped)
17	70.0, CH	5.47, m	70.1, CH	5.50, m	70.0, CH	5.53, m
18	19.9, CH_3_	1.28, d (6.2)	20.0, CH_3_	1.31, d (6.4)	19.9, CH_3_	1.33, d (6.3)
19	171.5, C		171.6, C		171.5, C	
20	105.8, C		106.0, C		106.0, C	
21	166.2, C		166.3, C		166.1, C	
22	102.7, CH	6.15 (overlapped)	102.9, CH	6.20 (overlapped)	102.7, CH	6.19 (overlapped)
23	163.4, C		163.5, C		163.6, C	
24	113.8, CH	6.16 (overlapped)	113.9, CH	6.20 (overlapped)	113.8, CH	6.20 (overlapped)
25	143.7, C		143.7, C		143.4, C	
26	43.5, CH_2_	3.23, dd (13.5, 3.9); 3.16, dd (13.5, 4.0)	43.6, CH_2_	3.20, dd (13.5, 3.7); 2.72 (overlapped)	43.6, CH_2_	3.21 (overlapped); 3.18 (overlapped)
27	72.9, CH	5.11, m	73.0, CH	5.15, m	72.9, CH	5.13, m
28	20.5, CH_3_	1.20, d (6.3)	20.6, CH_3_	1.24, d (6.2)	20.4, CH_3_	1.22, d (6.1)
29	171.9, C		171.5, C		171.9, C	
30	40.0, CH_2_	2.28, m; 2.33, m	36.8, CH_2_	2.57, dd (16.1, 4.8); 2.47, dd (16.1, 8.4)	40.0, CH_2_	2.43, dd (15.4, 5.9); 2.35, dd (15.4, 8.6)
31	67.0, CH	4.07, m	72.4, CH	5.14, m	67.0, CH	4.11, m
32	68.3, CH_2_	3.90 (overlapped)	63.6, CH_2_	3.57, dd (12.0, 4.2); 3.50, dd (12.0, 5.2)	68.4, CH_2_	3.94 (overlapped)
33	172.6, C		172.3, C		172.6, C	
34	20.7, CH_3_	1.99, s	21.0, CH_3_	1.96, s	20.7, CH_3_	2.04, s
1-OCH_3_					52.3, CH_3_	3.66, s

^a^ measured with 100 MHz for ^13^C and 400 MHz for ^1^H; ^b^ measured with 125 MHz for ^13^C and 500 MHz for ^1^H; ^c^ measured with 125 MHz for ^13^C and 400 MHz for ^1^H, ^d^ deduced from HMBC.

**Table 2:** *In vitro* antifungal activity (inhibition rates and IC_50_ values) of compounds **4**–**8** against *F. oxysporum* f. sp. *cubense*.

Compound	Concentration (μg/mL)	Inhibition Rate ± SD (%) (*n* = 3)	IC_50_ (μg/mL)
**4**	160	100	9.72
	80	100	
	40	96.32 ± 1.02	
	20	62.50 ± 1.62	
	10	51.47 ± 1.28	
**5**	160	100	21.07
	80	91.91 ± 1.25	
	40	69.85 ± 0.89	
	20	38.97 ± 1.43	
	10	34.55 ± 1.18	
**6**	160	100	7.89
	80	100	
	40	96.32 ± 0.82	
	20	70.59 ± 1.36	
	10	66.17 ± 1.23	
**7**	160	100	8.91
	80	100	
	40	96.32 ± 0.91	
	20	69.12 ± 1.16	
	10	61.76 ± 0.93	
**8**	160	100	9.65
	80	100	
	40	92.65 ± 0.69	
	20	67.64 ± 0.85	
	10	55.88 ± 1.42	
Ketoconazole	160	100	46.23
	80	58.82 ± 0.58	
	40	46.32 ± 0.76	
	20	42.64 ± 1.08	
	10	35.29 ± 1.63	

**Table 3:** Molecular docking interaction of isolated compounds with the crystal structure of *F. oxysporum* (PDB 1FN8).

Compound	Binding Energy (kcal/mol)	Hydrogen Bonds with Residues (Bond Lengths, Å)	Hydrophobic Contacts with Residues
**1**	−3.35	ND	ND
**2**	−1.97	ND	ND
**3**	−3.57	ND	ND
**4**	−9.53	ASN99 (1.90, 3.02), GLY219 (2.78, 3.11), Tyr0 (3.48), Tyr94 (2.82)	Ala175, Tyr172, Asn217, Arg0, Gly216, Trp215, His57, Gly96
**5**	−9.01	Arg122 (2.51), Ala125 (3.17), Arg235 (2.82, 3.28)	Ser202, Thr204, Ser134, Ile210, Ser0, Pro130, Asp201, Asp129, Ser128, Leu123, Ala124
**6**	−9.94	Tyr94 (3.04), Val90 (2.70), Gln60 (2.96), (3.16)	Pro92, Ser0, Arg89, Ser86, Val88, Ser87
**7**	−12.25	Tyr94 (3.10), Val90 (2.62), Tyr0 (3.27, 3.24), Gln60 (2.82)	Pro92, Arg89, Val58, Ser0, Ser87, Val59
**8**	−9.41	Val90 (2.61), His91 (3.00)	Pro92, Gly96, Ser95, Tyr94, Ser0, Gly0, Val88, Arg89, Gln60
**9**	−4.12	ND	ND
**10**	−1.96	ND	ND
**11**	−5.90	ND	ND
**12**	−5.94	ND	ND
**13**	−4.51	ND	ND
**14**	−6.72	ND	ND
**15**	−6.85	ND	ND
**16**	−7.42	ND	ND
Benzamidine	−6.80	Pro28 (2.1), Ser70 (2.0), Ile30 (2.1, 2.0)	Ser70, Phe27, Leu155, Leu71, Gly25, Ser23, Asp26, Asn117, Gly69

ND: not determined.

## Data Availability

The original contributions presented in this study are included in the article/Supplementary Materials. Further inquiries can be directed to the corresponding authors.

## Supplementary Materials

The following supporting information can be downloaded at: https://www.mdpi.com/article/10.3390/biology15120920/s1, Figure S1: HRESIMS spectrum of **1**; Figure S2: ^1^H NMR spectrum of compound **1**; Figure S3: ^13^C NMR spectrum of compound **1**; Figure S4: ^1^H–^1^H COSY spectrum of compound **1**; Figure S5: HSQC spectrum of compound **1**; Figure S6: HMBC spectrum of compound **1**; Figure S7: HRESIMS spectrum of **2**; Figure S8: ^1^H NMR spectrum of compound **2**; Figure S9: ^13^C NMR spectrum of compound **2**; Figure S10: ^1^H–^1^H COSY spectrum of compound **2**; Figure S11: HSQC spectrum of compound **2**; Figure S12: HMBC spectrum of compound **2**; Figure S13: HRESIMS spectrum of **3**; Figure S14: ^1^H NMR spectrum of compound **3**; Figure S15: ^13^C NMR spectrum of compound **3**; Figure S16: ^1^H–^1^H COSY spectrum of compound **3**; Figure S17: HSQC spectrum of compound **3**; Figure S18: HMBC spectrum of compound **3**; Figure S19: ^1^H NMR spectrum of compound **4**; Figure S20: ^13^C NMR spectrum of compound **4**; Figure S21: ^1^H NMR spectrum of compound **5**; Figure S22: ^13^C NMR spectrum of compound **5**; Figure S23: ^1^H NMR spectrum of compound **6**; Figure S24: ^13^C NMR spectrum of compound **6**; Figure S25: ^1^H NMR spectrum of compound **7**; Figure S26: ^13^C NMR spectrum of compound **7**; Figure S27: ^1^H NMR spectrum of compound **8**; Figure S28: ^13^C NMR spectrum of compound **8**; Figure S29: ^1^H NMR spectrum of compound **9**; Figure S30: ^13^C NMR spectrum of compound **9**; Figure S31: ^1^H NMR spectrum of compound **10**; Figure S32: ^13^C NMR spectrum of compound **10**; Figure S33: ^1^H NMR spectrum of compound **11**; Figure S34: ^13^C NMR spectrum of compound **11**; Figure S35: ^1^H NMR spectrum of compound **12**; Figure S36: ^13^C NMR spectrum of compound **12**; Figure S37: ^1^H NMR spectrum of compound **13**; Figure S38: ^13^C NMR spectrum of compound **13**; Figure S39: ^1^H NMR spectrum of compound **14**; Figure S40: ^13^C NMR spectrum of compound **14**; Figure S41: ^1^H NMR spectrum of compound **15**; Figure S42: ^13^C NMR spectrum of compound **15**; Figure S43: ^1^H NMR spectrum of compound **16**; Figure S44: ^13^C NMR spectrum of compound **16**.
