# Supplementary material for: Study on the Antifungal Activity and Molecular Docking of Polyester Metabolites from Talaromyces striatoconidius
Source: Biology (Basel). 2026 Jun 12;15(12):920. doi: 10.3390/biology15120920 (PMC13295287; doi:10.3390/biology15120920)
Supplement: Supplementary file 1 [file biology-15-00920-s001.zip › biology-4365471-supplementary.pdf]

Antifungal activity and molecular docking study of polyester metabolites from *Talaromyces striatoconidius*

Yanyan Chen 1, Mingjie Zhang 1, Siqin Li 1, Jiekang Xiao 1, Zheng Ma 2, Jiawen Sun 3, Xiachang Wang 3, Junwei Sun 4,* and Yongyong Zhang 1,5,*

1 Department of Pharmacy, College of Life Sciences, China Jiliang University, Hangzhou 310018, China; p24091055007@cjlu.edu.cn (Y.C.); 2300903101@cjlu.edu.cn (M.Z.); 2300903131@cjlu.edu.cn (S.L.); 17870235892@163.com (J.X.)

2 Zhejiang Provincial Key Laboratory of Biometrology and Inspection & Quarantine, College of Life Sciences, China Jiliang University, Hangzhou 310018, China; mazheng520@163.com

3 Jiangsu Key Laboratory for Functional Substances of Chinese Medicine, Nanjing University of Chinese Medi-cine, Nanjing 210023, China; 20230867@njucm.edu.cn (J.S.); xiachangwang@njucm.edu.cn (X.W.)

4 College of Modern Science and Technology, China Jiliang University, Yiwu 322002, China

5 Key Laboratory of Marine Food Quality and Hazard Controlling Technology of Zhejiang Province, College of Life Sciences, China Jiliang University, Hangzhou 310018, China

* Correspondence: juville@cjlu.edu.cn (J.S.); zhangyy76@163.com (Y.Z.)

Contents

Figure S1. HRESIMS spectrum of compound **1**.

Figure S2. ^{1}H NMR spectrum of compound **1**.

Figure S3. ^{13}C NMR spectrum of compound **1**.

Figure S4. ^{1}H-^{1}H COSY spectrum of compound **1**.

Figure S5. HSQC spectrum of compound **1**.

Figure S6. HMBC spectrum of compound **1**.

Figure S7. HRESIMS spectrum of compound **2**.

Figure S8. ^{1}H NMR spectrum of compound **2**.

Figure S9. ^{13}C NMR spectrum of compound **2**.

Figure S10. ^{1}H-^{1}H COSY spectrum of compound **2**.

Figure S11. HSQC spectrum of compound **2**.

Figure S12. HMBC spectrum of compound **2**.

Figure S13. HRESIMS spectrum of compound **3**.

Figure S14. ^{1}H NMR spectrum of compound **3**.

Figure S15. ^{13}C NMR spectrum of compound **3**.

Figure S16. ^{1}H-^{1}H COSY spectrum of compound **3**.

Figure S17. HSQC spectrum of compound **3**.

Figure S18. HMBC spectrum of compound **3**.

Figure S19. ^{1}H NMR spectrum of compound **4**.

Figure S20. ^{13}C NMR spectrum of compound **4**.

Figure S21. ^{1}H NMR spectrum of compound **5**.

Figure S22. ^{13}C NMR spectrum of compound **5**.

Figure S23. ^{1}H NMR spectrum of compound **6**.

Figure S24. ^{13}C NMR spectrum of compound **6**.

Figure S25. ^{1}H NMR spectrum of compound **7**.

Figure S26. ^{13}C NMR spectrum of compound **7**.

Figure S27. ^{1}H NMR spectrum of compound **8**.

Figure S28. ^{13}C NMR spectrum of compound **8**.

Figure S29. ^{1}H NMR spectrum of compound **9**.

Figure S30. ^{13}C NMR spectrum of compound **9**.

Figure S31. ^{1}H NMR spectrum of compound **10**.

Figure S32. ^{13}C NMR spectrum of compound **10**.

Figure S33. ^{1}H NMR spectrum of compound **11**.

Figure S34. ^{13}C NMR spectrum of compound **11**.

Figure S35. ^{1}H NMR spectrum of compound **12**.

Figure S36. ^{13}C NMR spectrum of compound **12**.

Figure S37. ^{1}H NMR spectrum of compound **13**.

Figure S38. ^{13}C NMR spectrum of compound **13**.

Figure S39. ^{1}H NMR spectrum of compound **14**.

Figure S40. ^{13}C NMR spectrum of compound **14**.

Figure S41. ^{1}H NMR spectrum of compound **15**.

Figure S42. ^{13}C NMR spectrum of compound **15**.

Figure S43. ^{1}H NMR spectrum of compound **16**.

Figure S44. ^{13}C NMR spectrum of compound **16**.

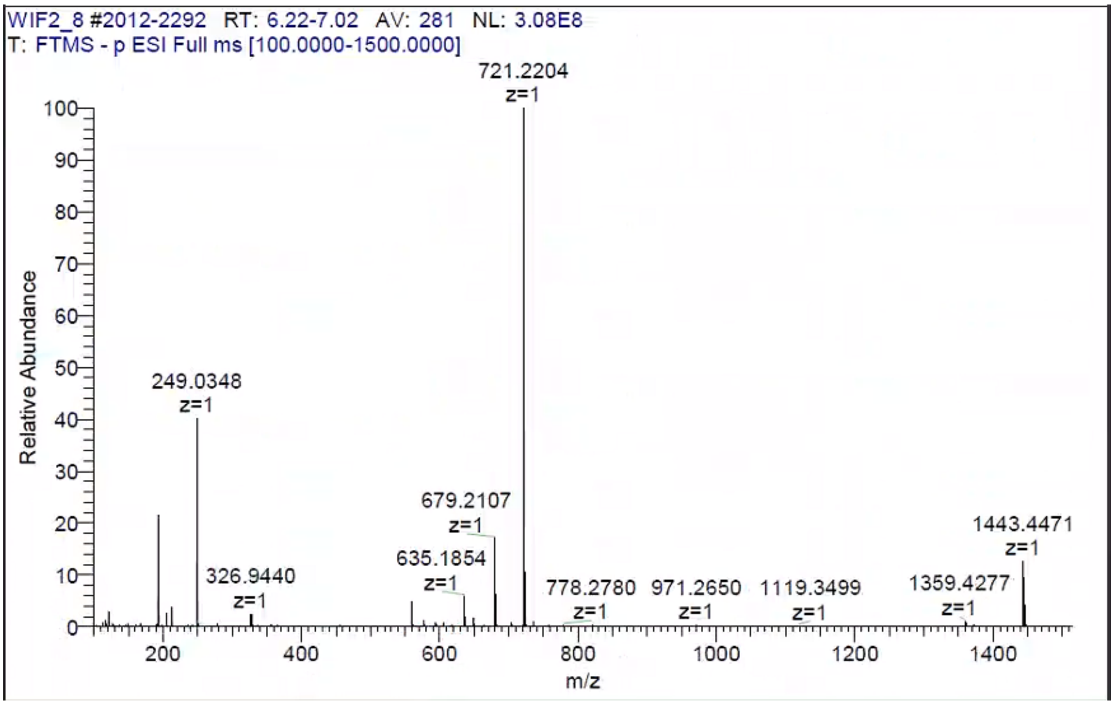

Figure S1. HRESIMS spectrum of compound **1**.

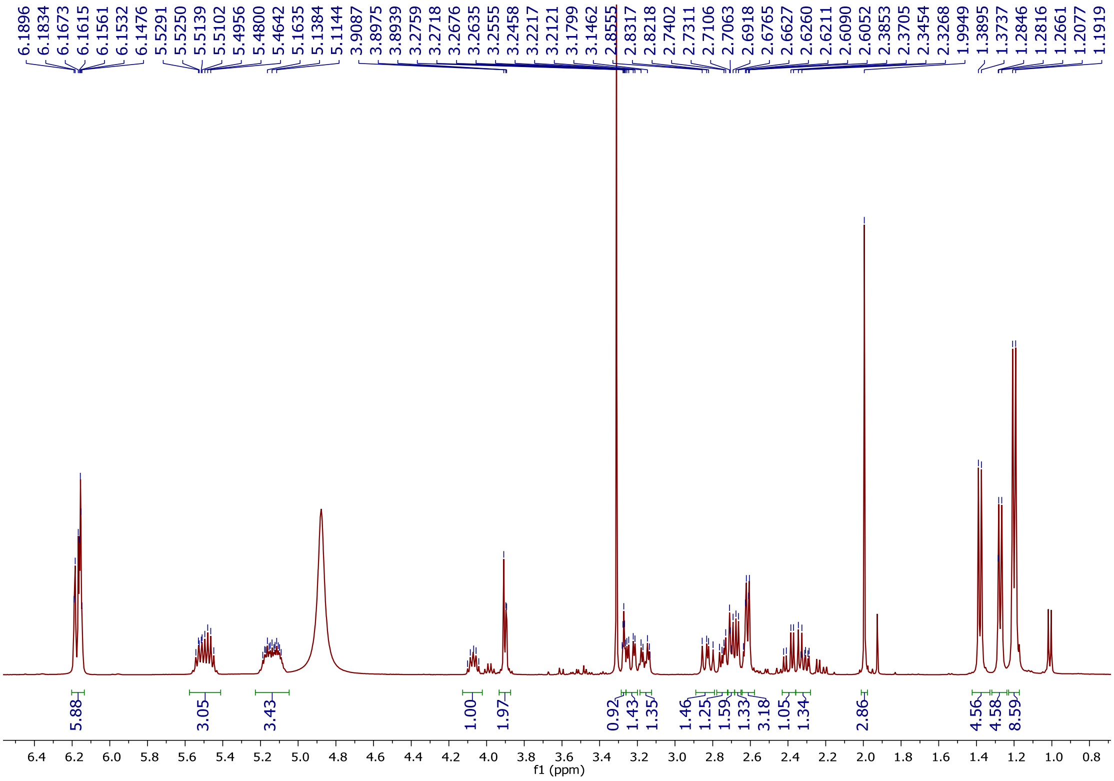

Figure S2. ^{1}H NMR spectrum of compound **1**.

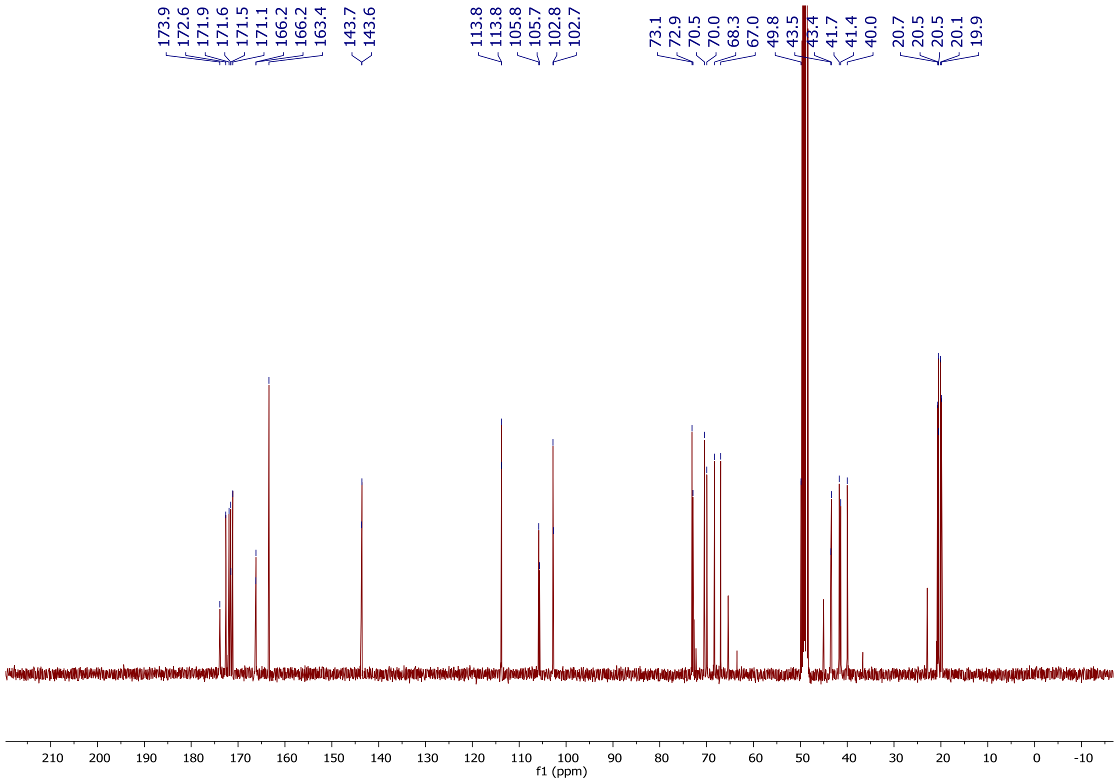

Figure S3. ^{13}C NMR spectrum of compound **1**.

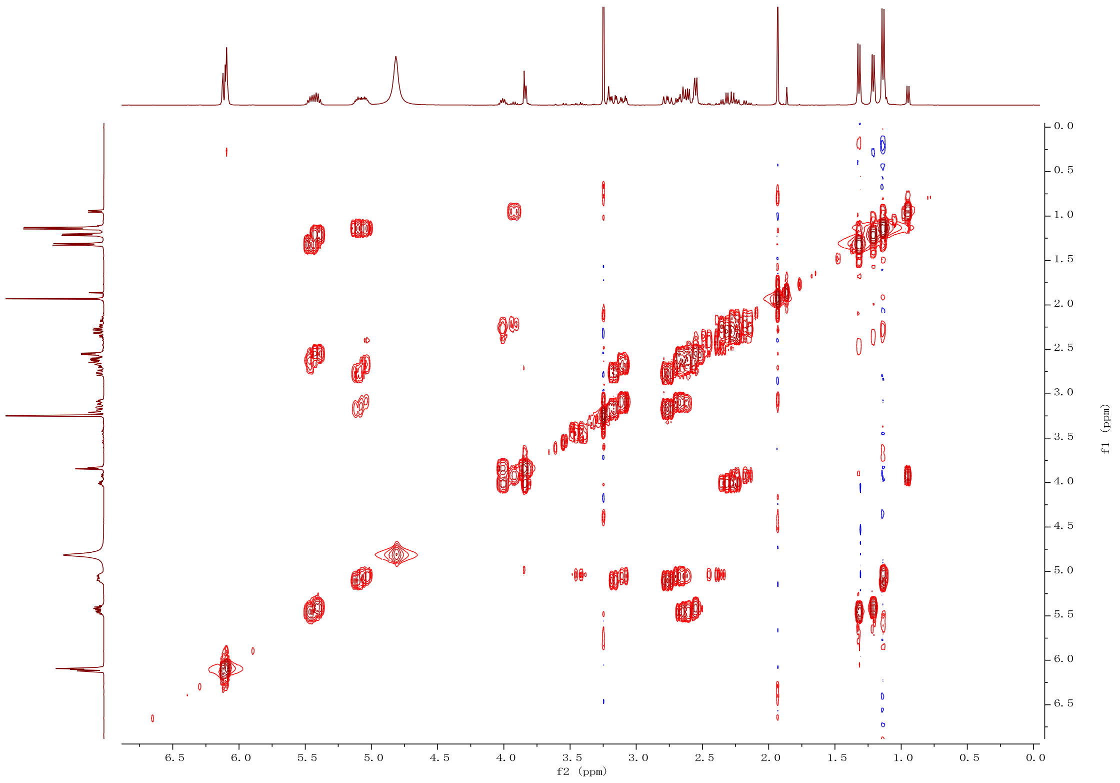

Figure S4. ^{1}H-^{1}H COSY spectrum of compound **1**.

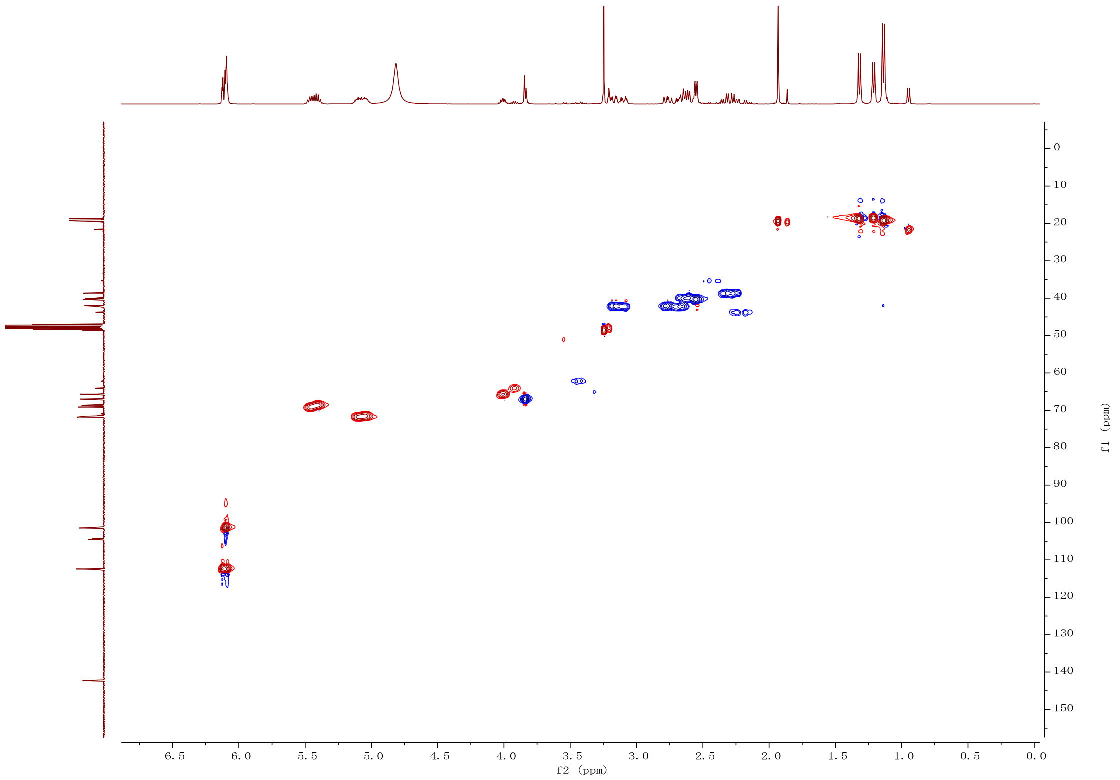

Figure S5. HSQC spectrum of compound **1**.

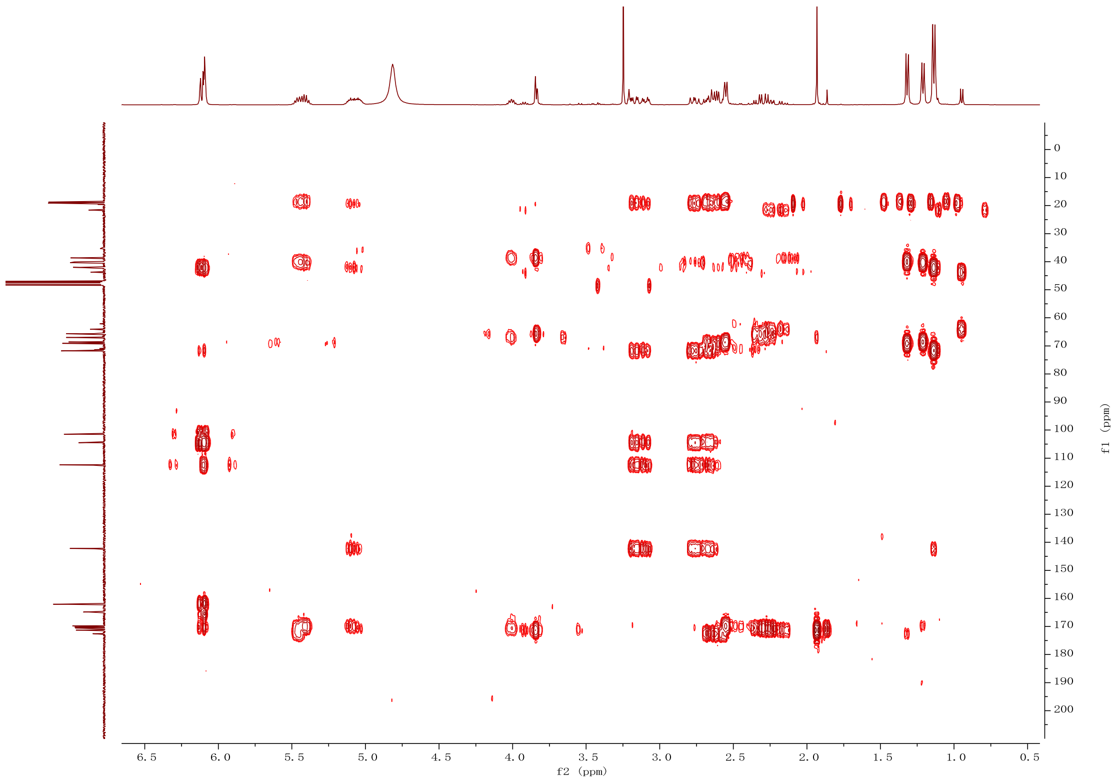

Figure S6. HMBC spectrum of compound **1**.

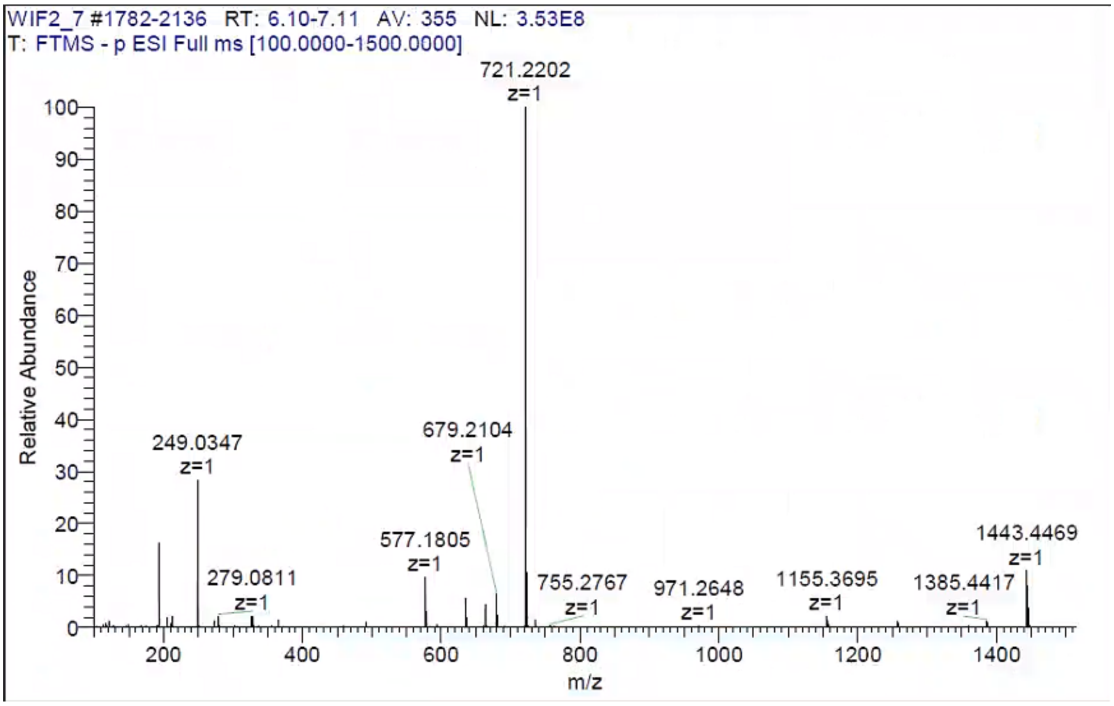

Figure S7. HRESIMS spectrum of compound **2**.

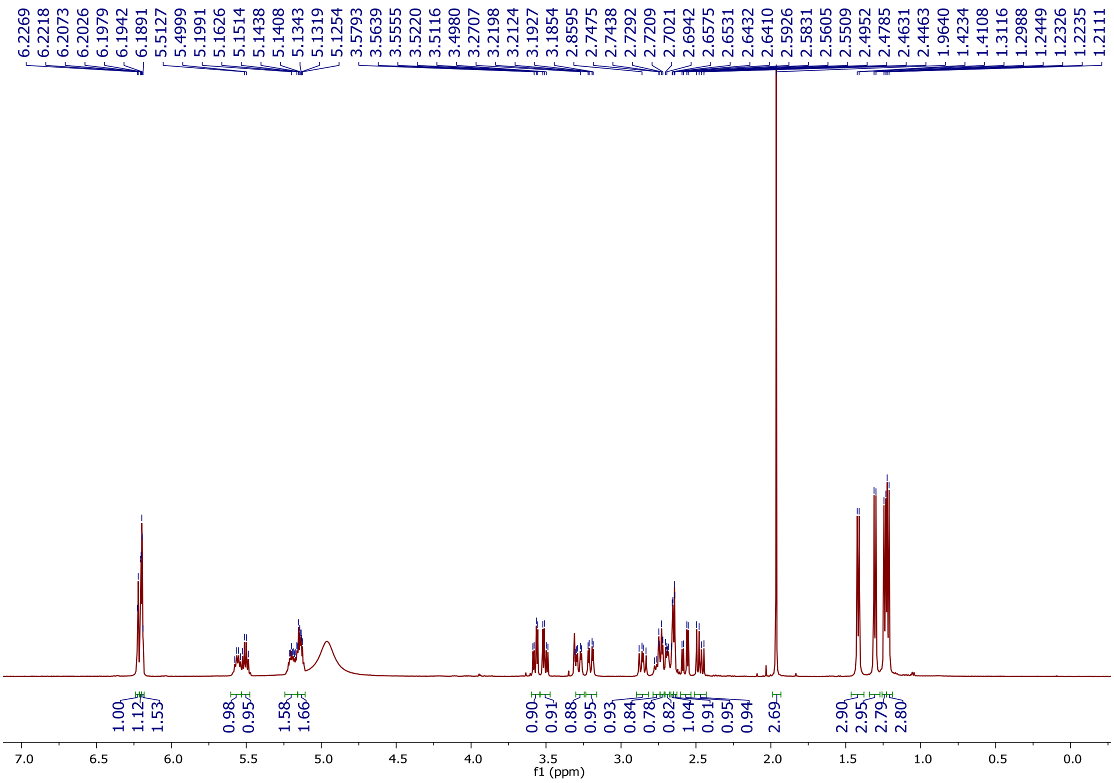

Figure S8. ^{1}H NMR spectrum of compound **2**.

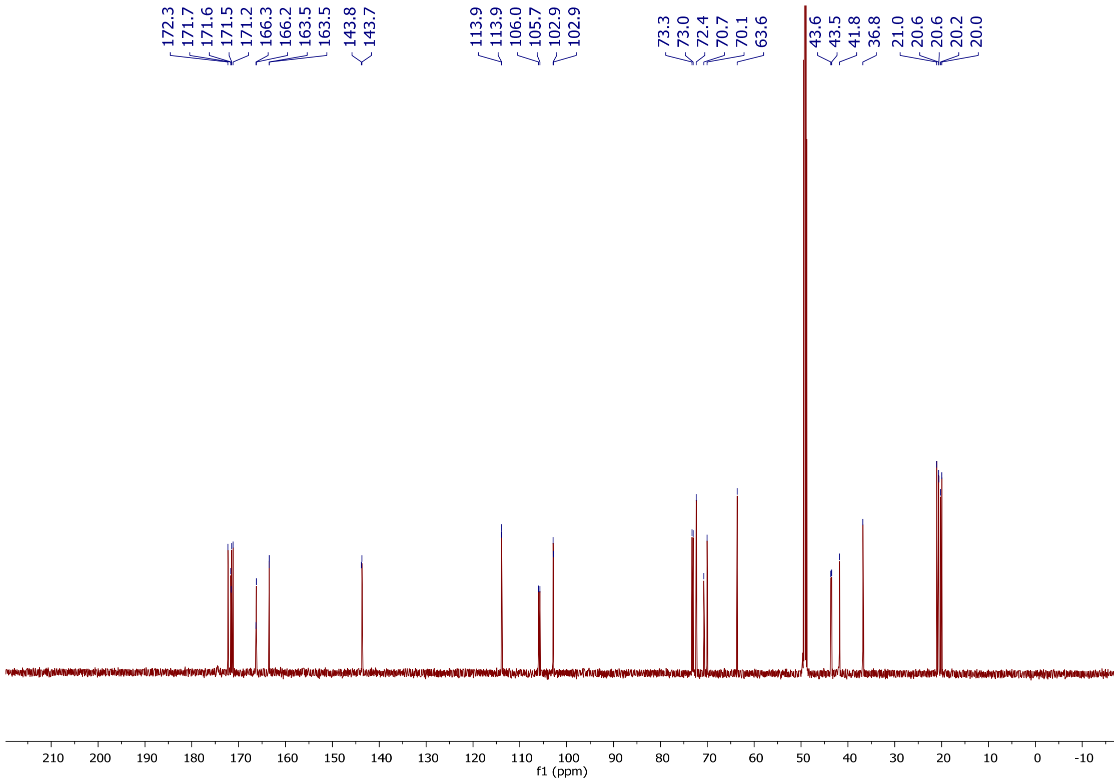

Figure S9. ^{13}C NMR spectrum of compound **2**.

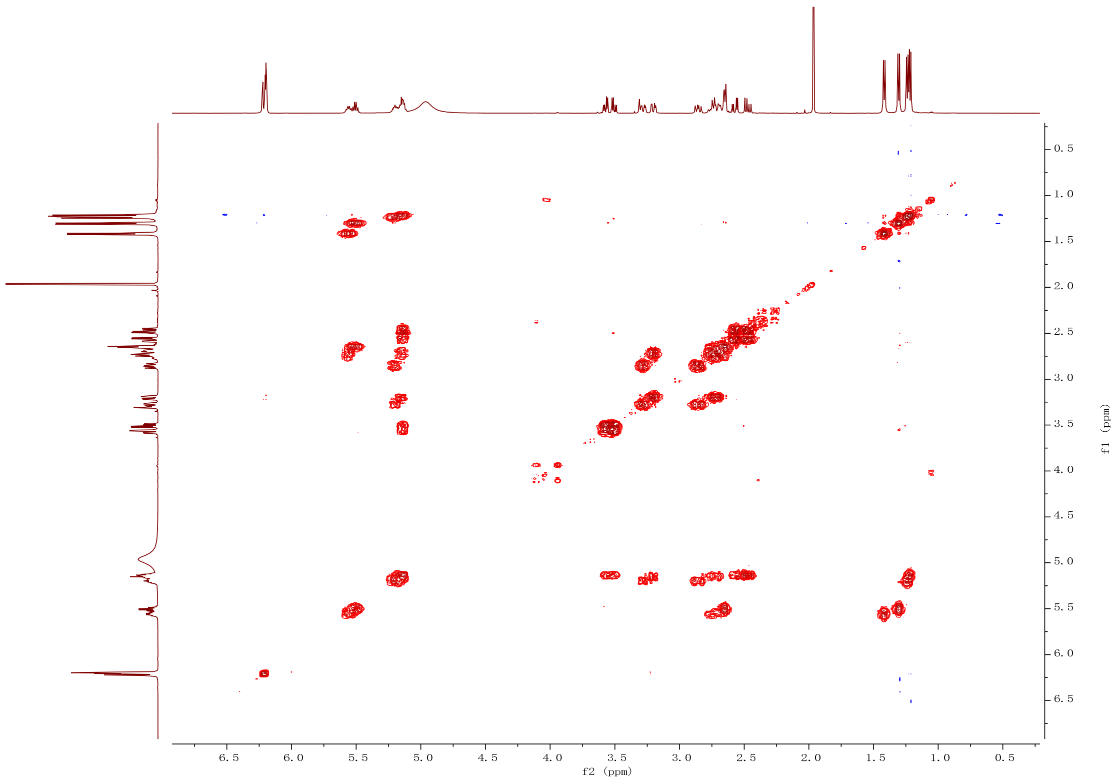

Figure S10. ^{1}H-^{1}H COSY spectrum of compound **2**.

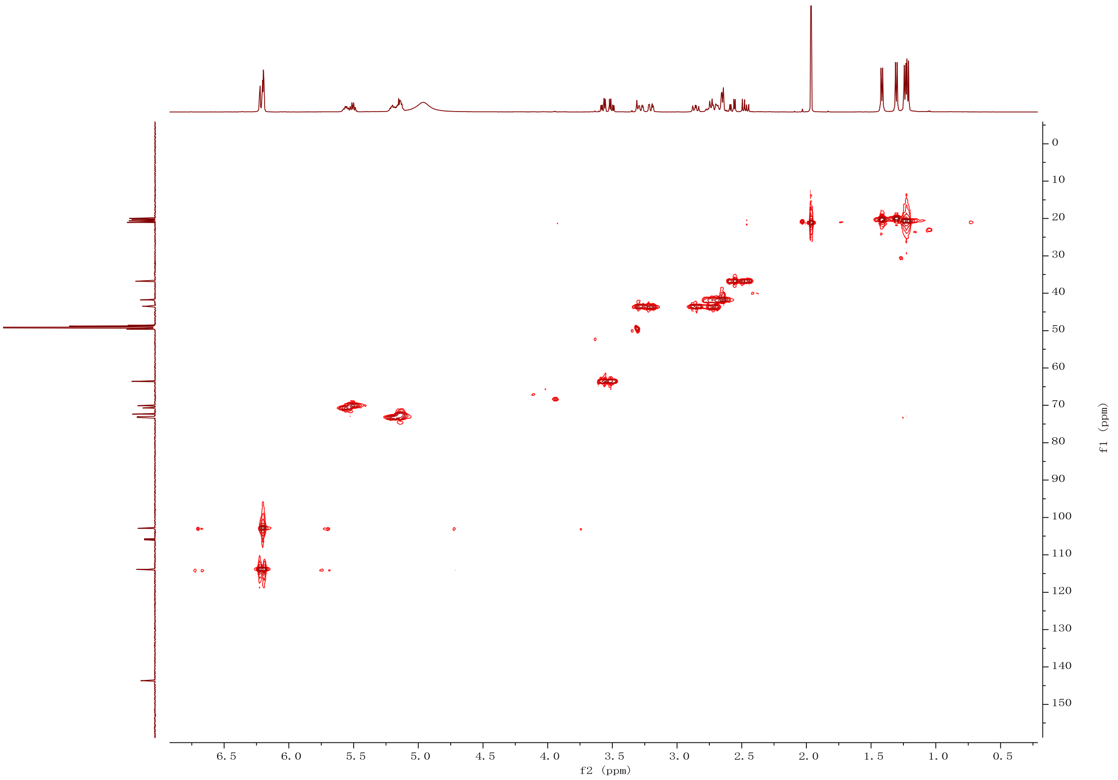

Figure S11. HSQC spectrum of compound **2**.

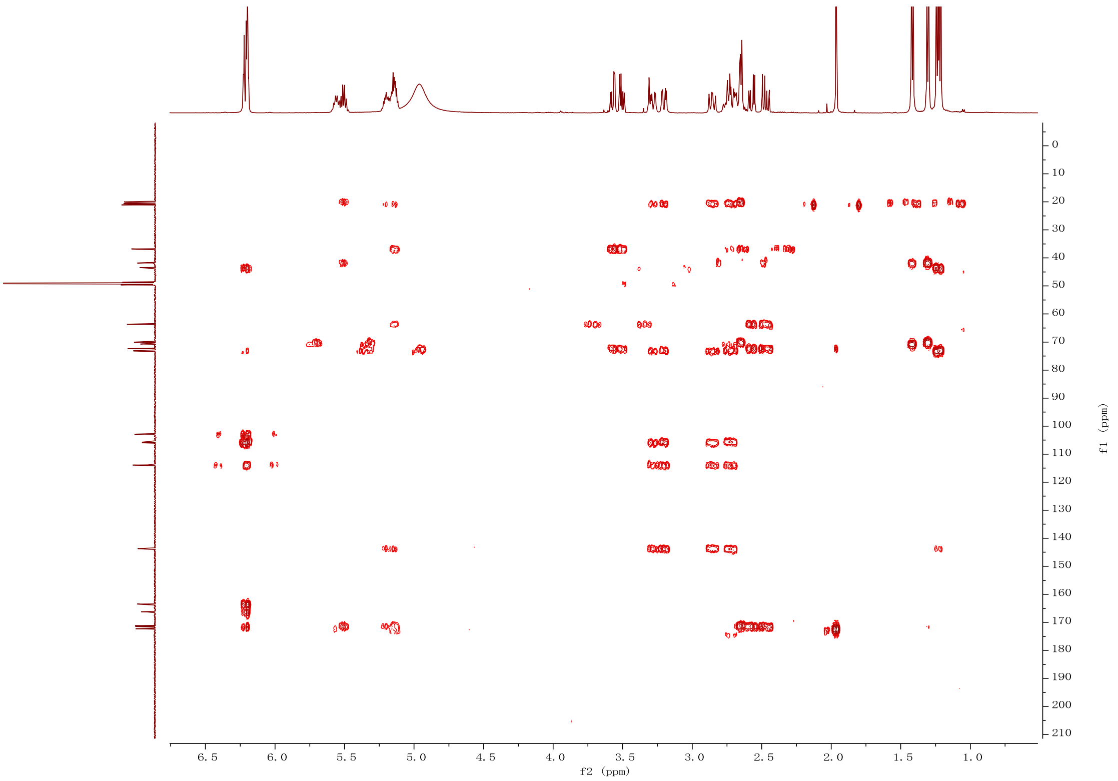

Figure S12. HMBC spectrum of compound **2**.

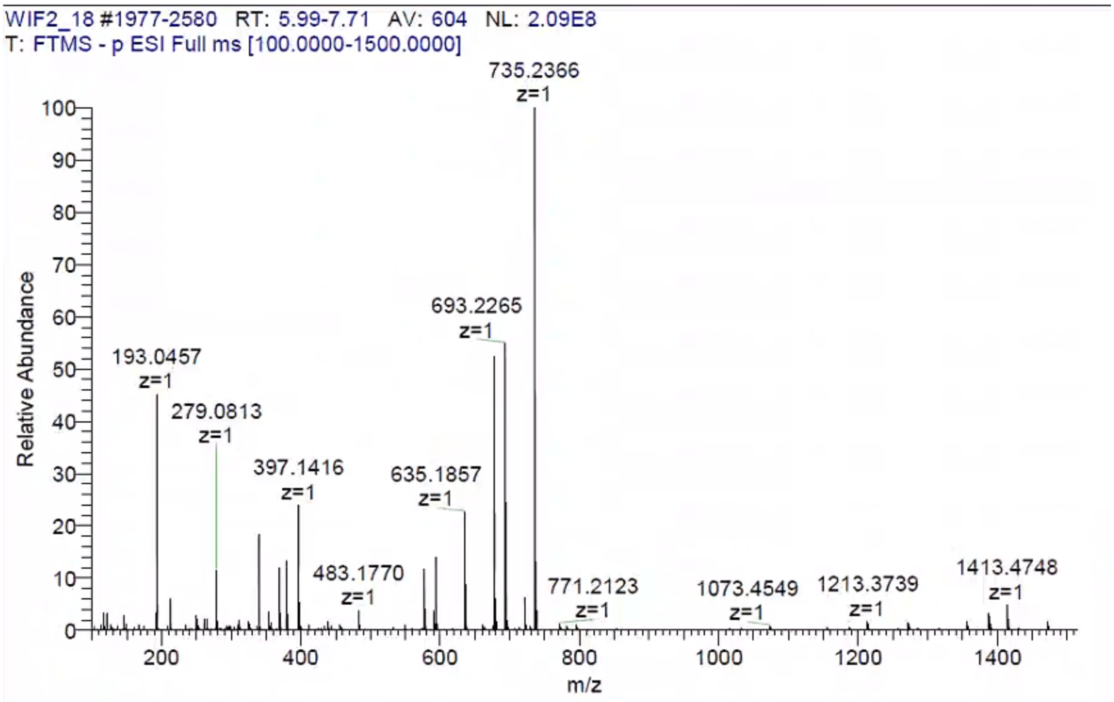

Figure S13. HRESIMS spectrum of compound **3**.

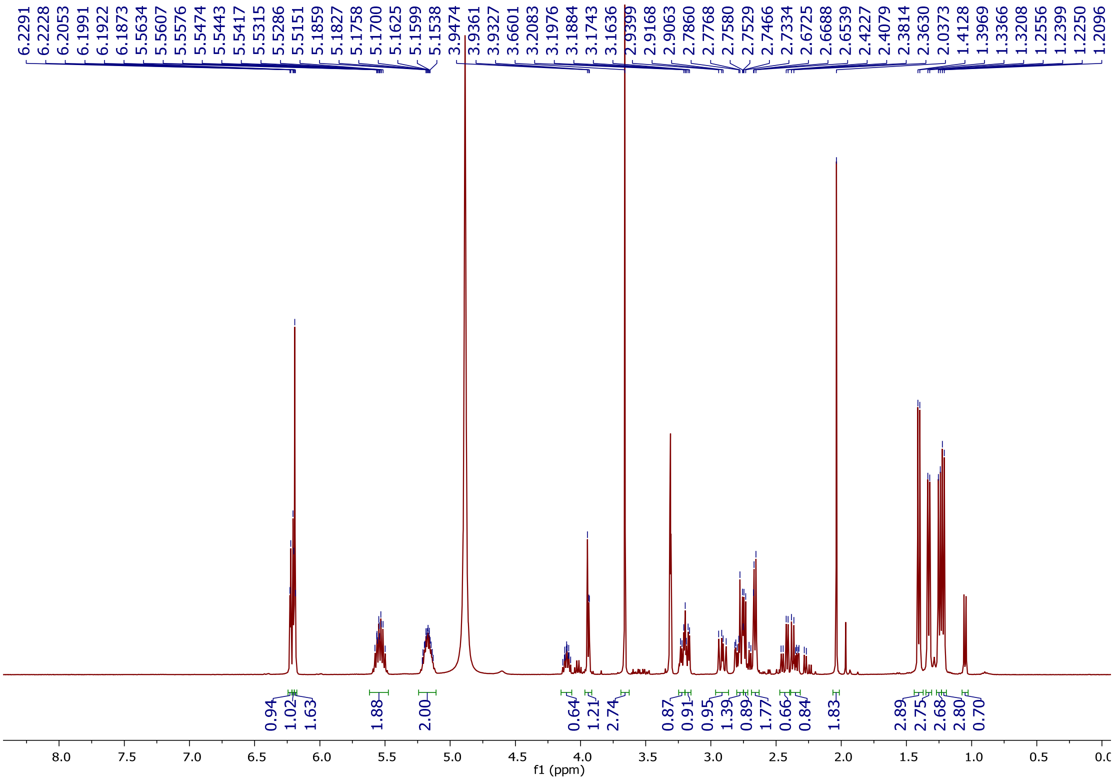

Figure S14. ^{1}H NMR spectrum of compound **3**.

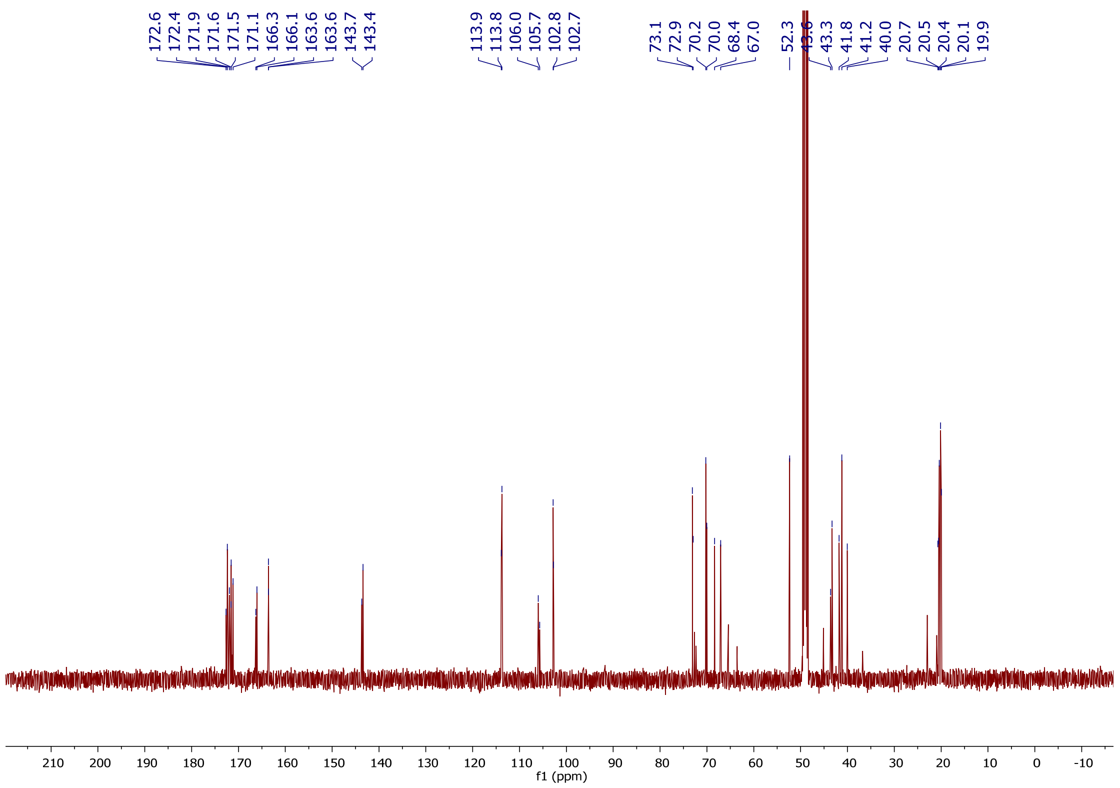

Figure S15. ^{13}C NMR spectrum of compound **3**.

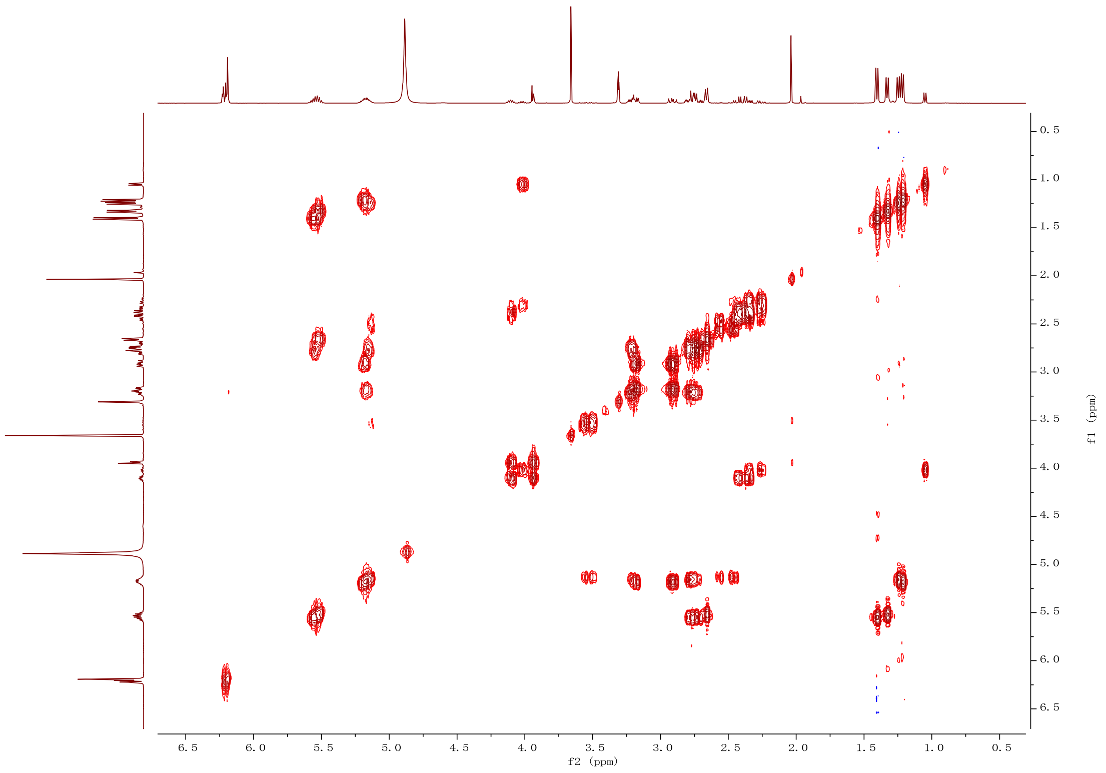

Figure S16. ^{1}H-^{1}H COSY spectrum of compound **3**.

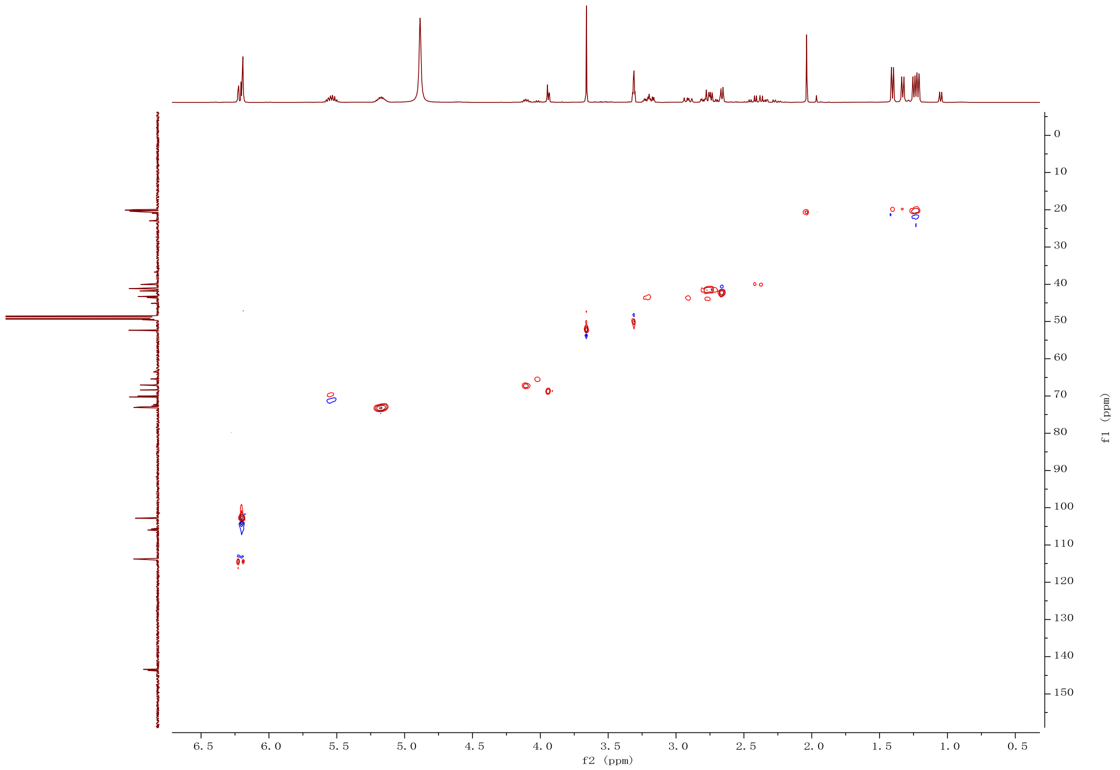

Figure S17. HSQC spectrum of compound **3**.

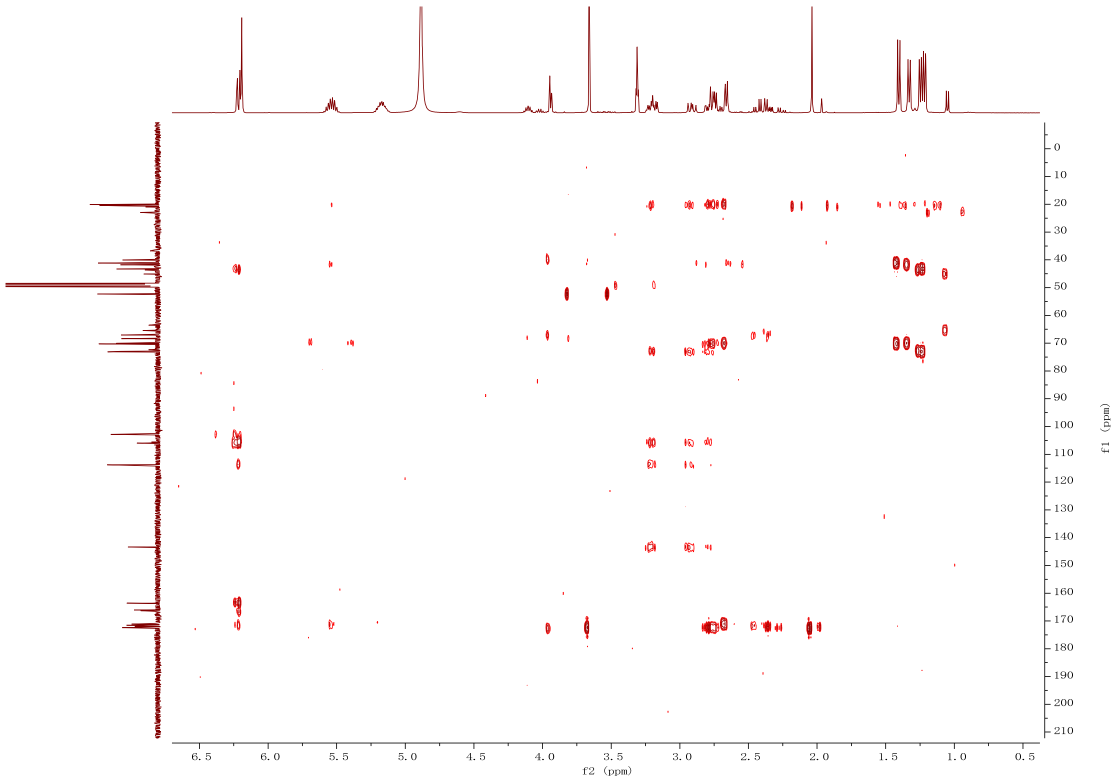

Figure S18. HMBC spectrum of compound **3**.

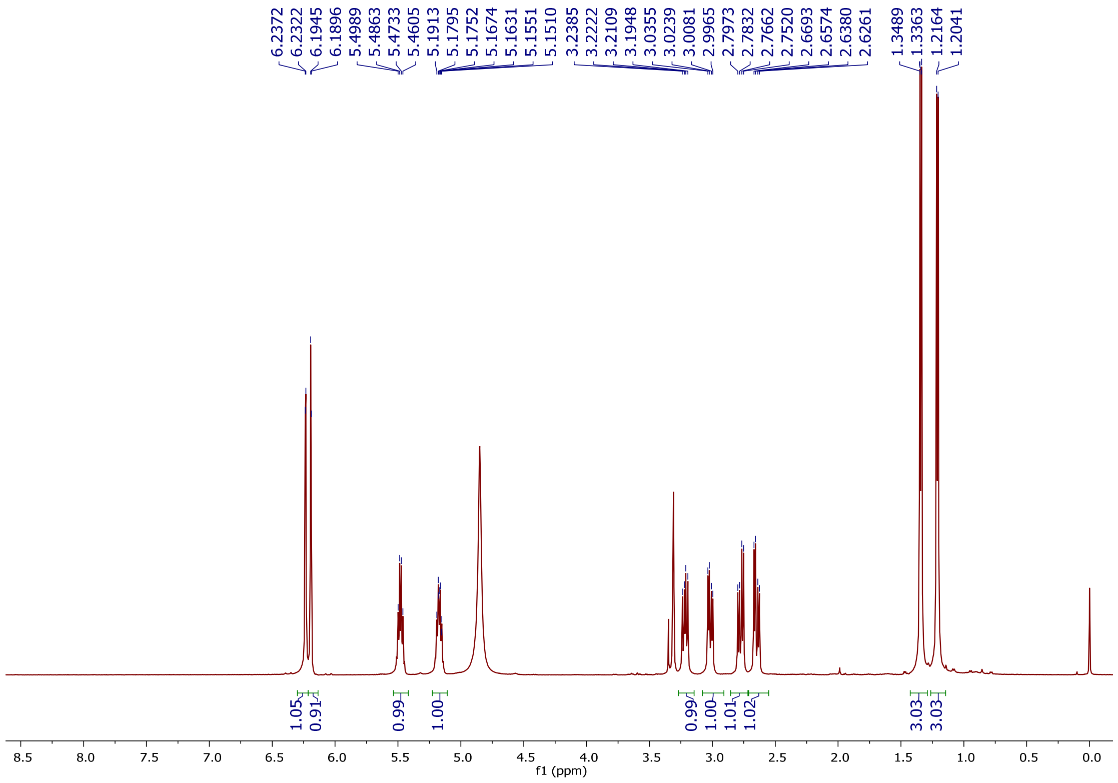

Figure S19. ^{1}H NMR spectrum of compound **4**.

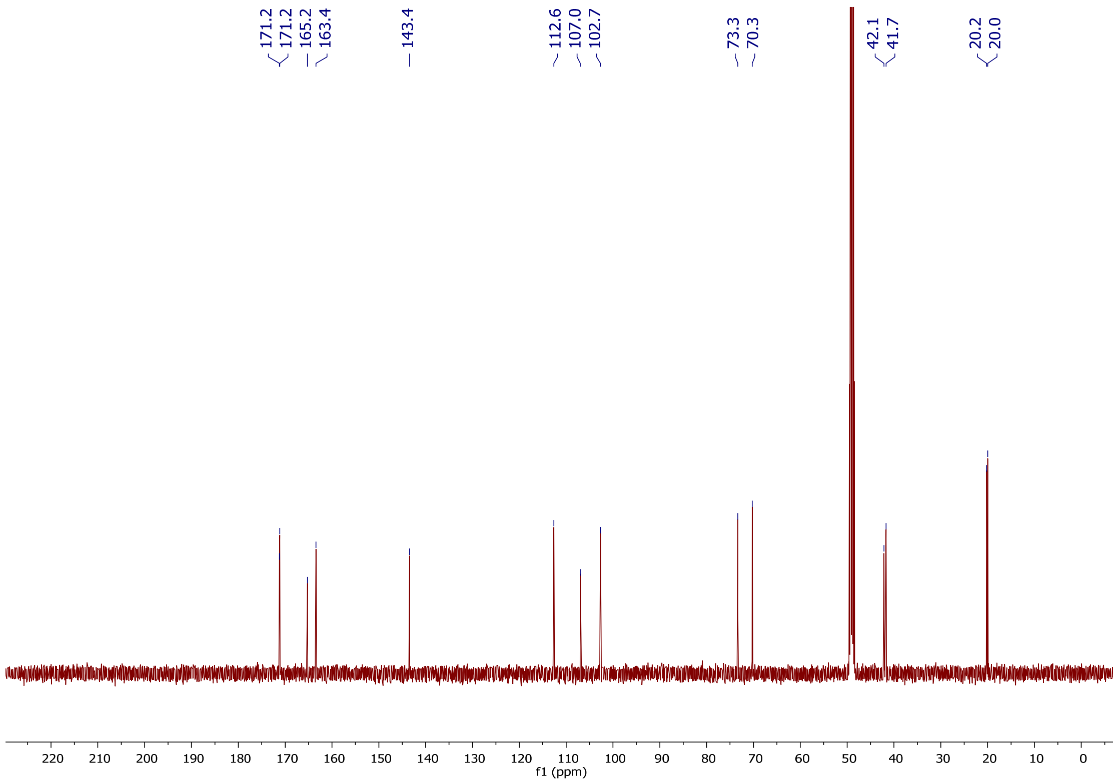

Figure S20. ^{13}C NMR spectrum of compound **4**.

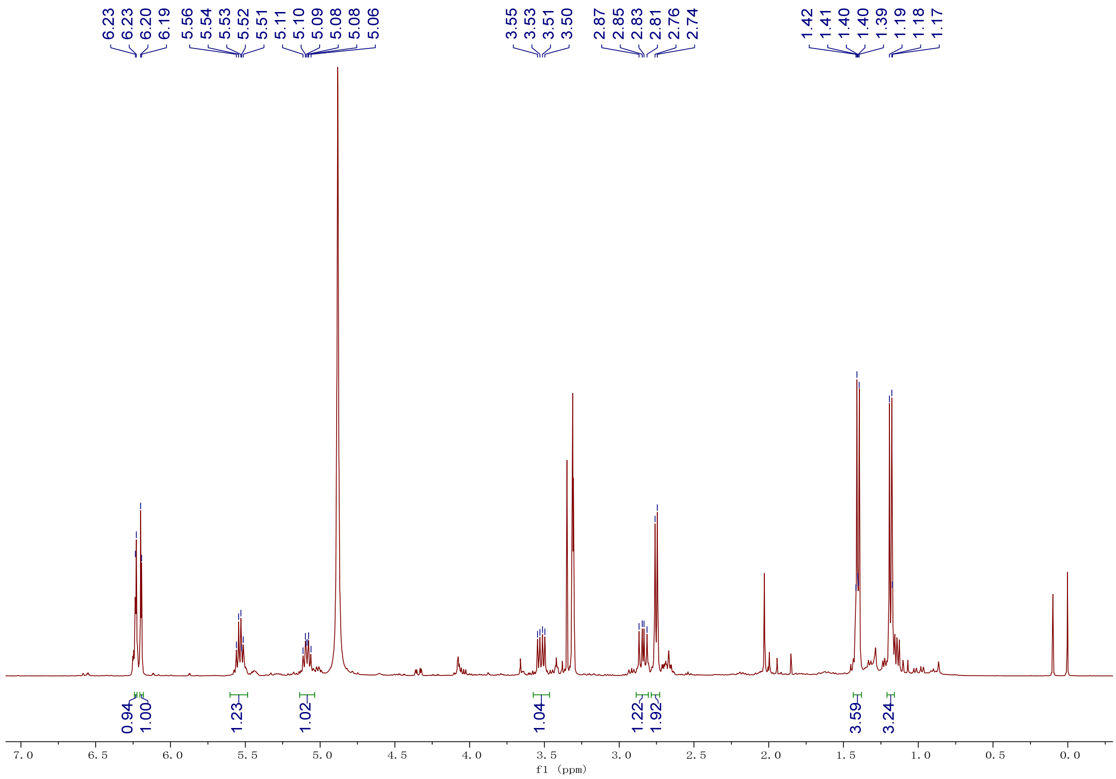

Figure S21. ^{1}H NMR spectrum of compound **5**.

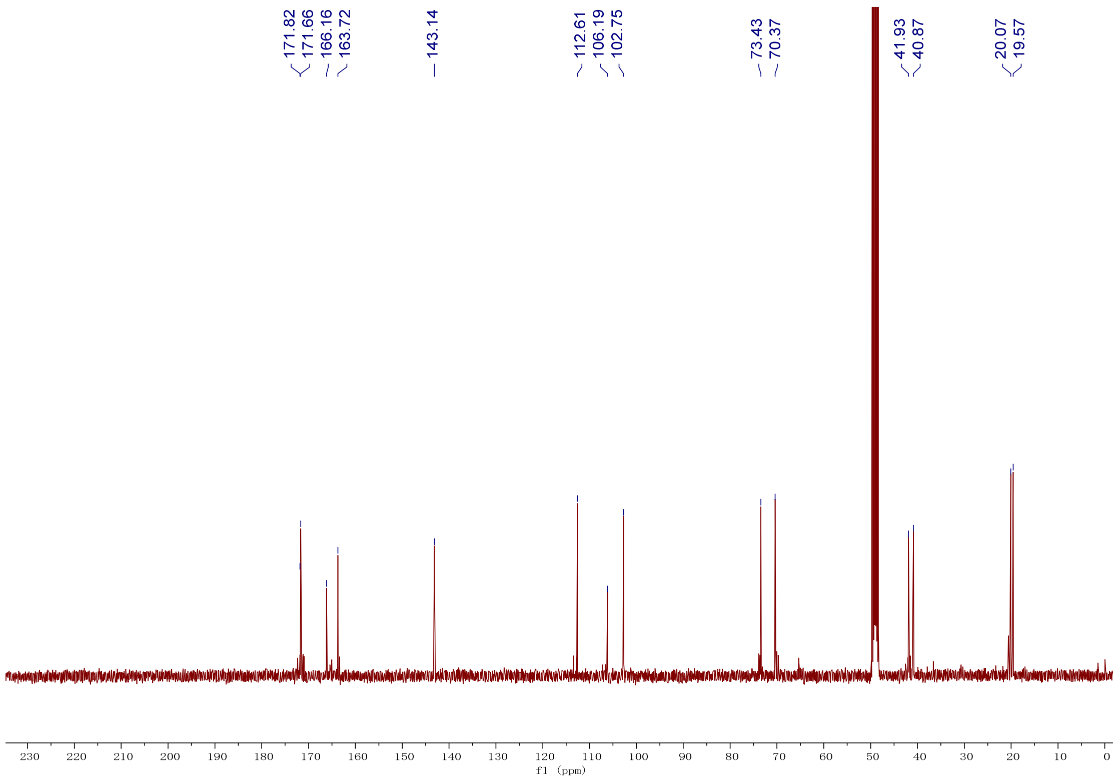

Figure S22. ^{13}C NMR spectrum of compound **5**.

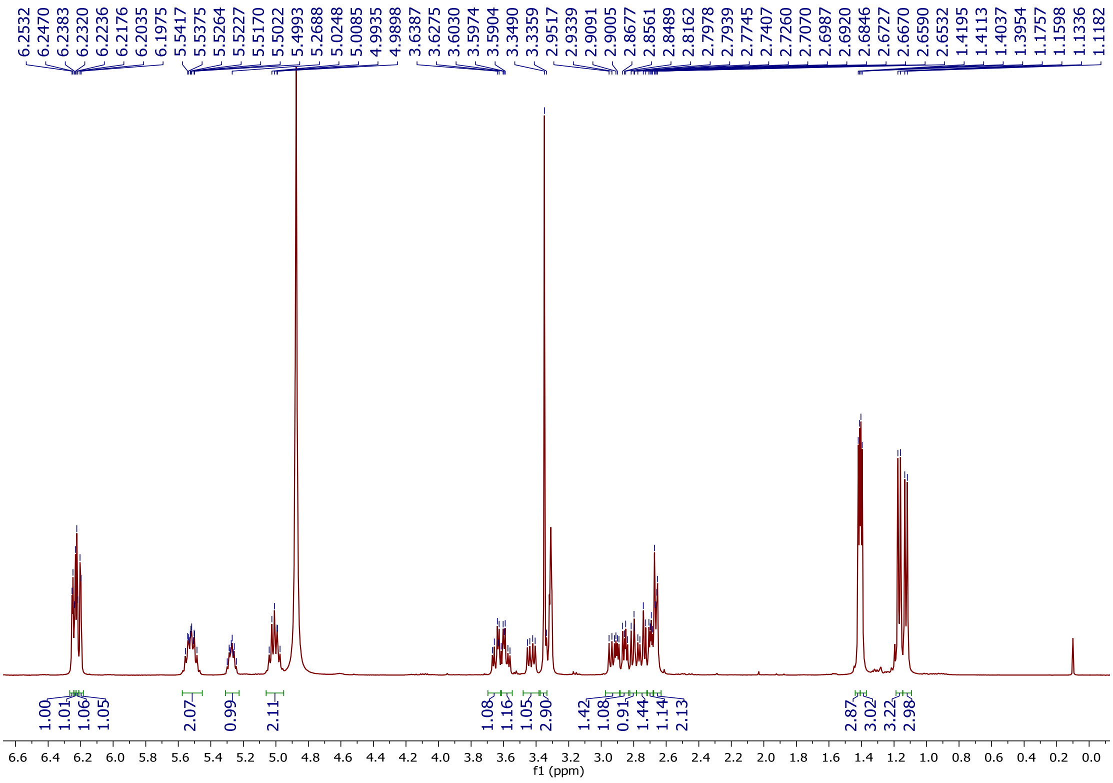

Figure S23. ¹H NMR spectrum of compound **6**.

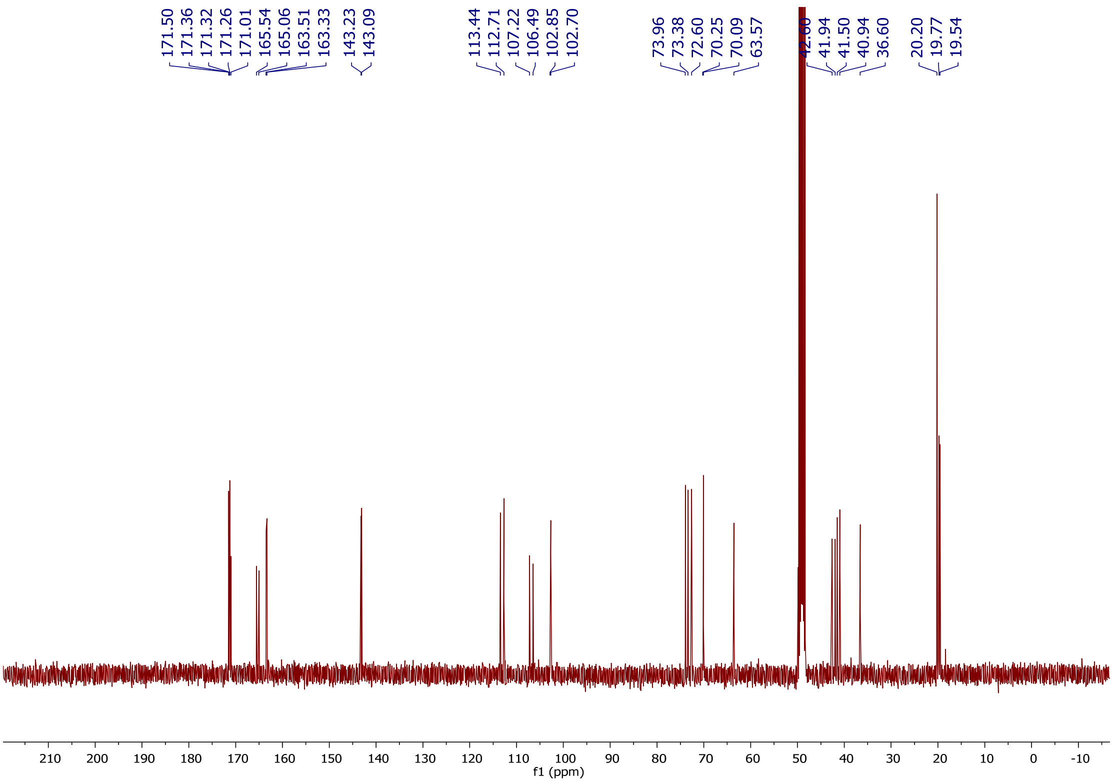

Figure S24. ¹³C NMR spectrum of compound **6**.

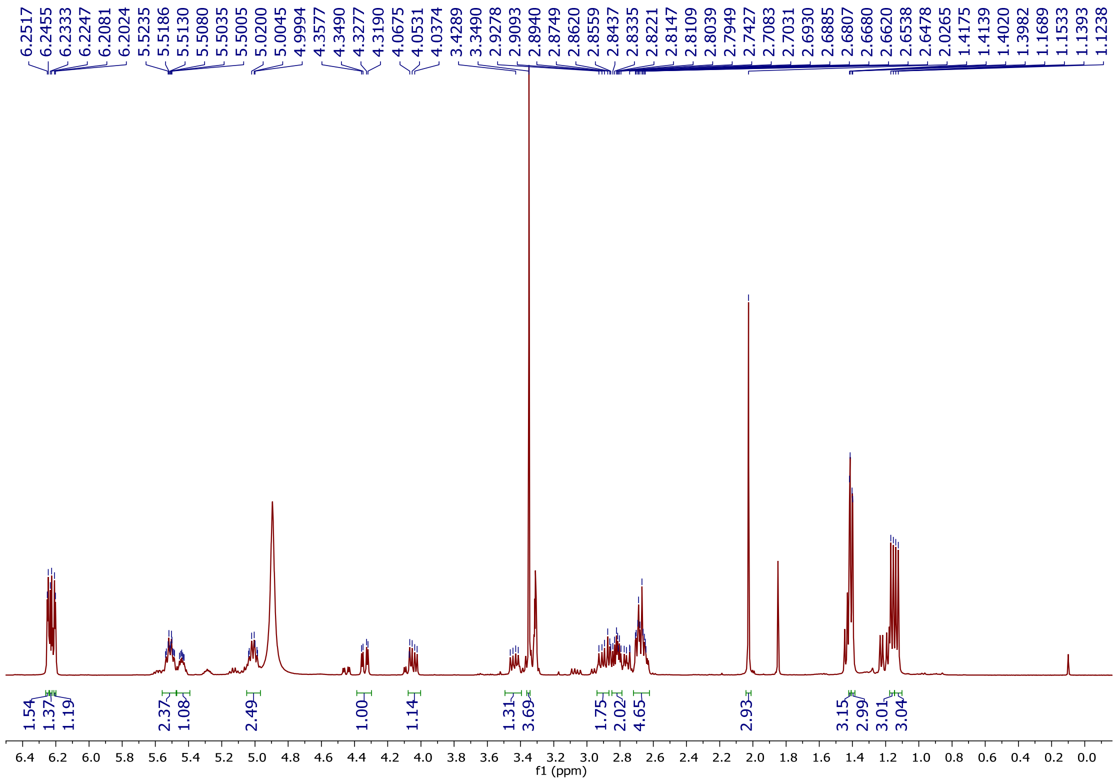

Figure S25. ^{1}H NMR spectrum of compound **7**.

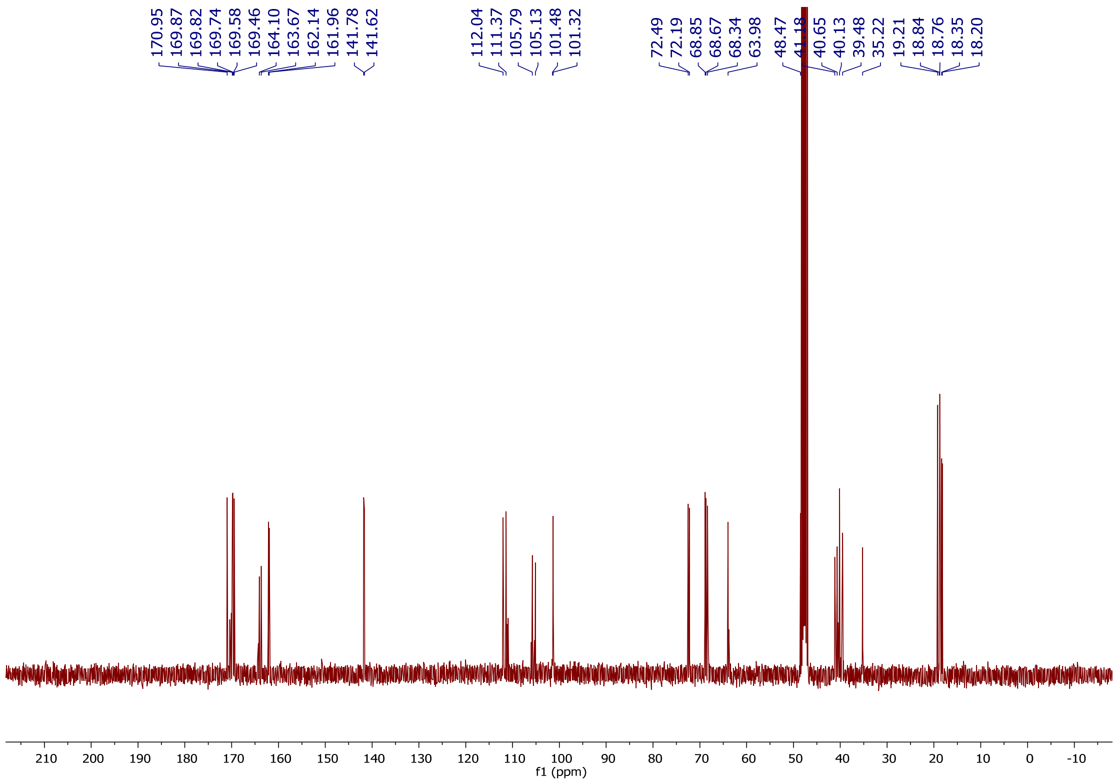

Figure S26. ^{13}C NMR spectrum of compound **7**.

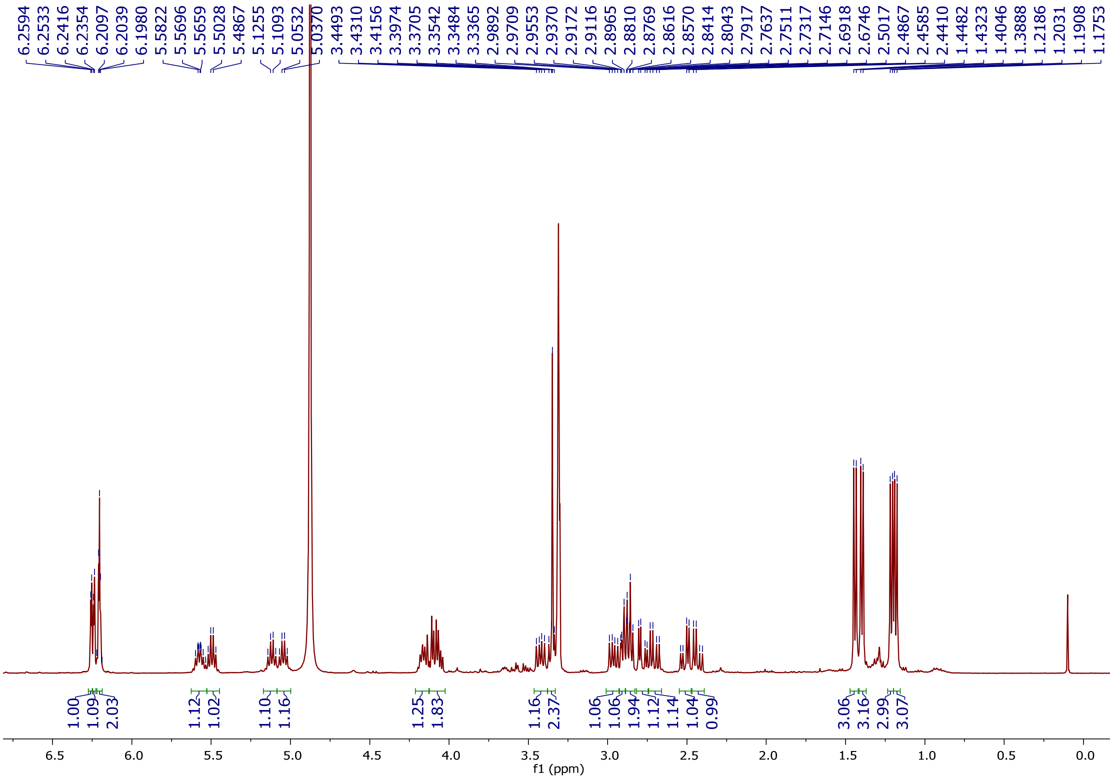

Figure S27. ^{1}H NMR spectrum of compound **8**.

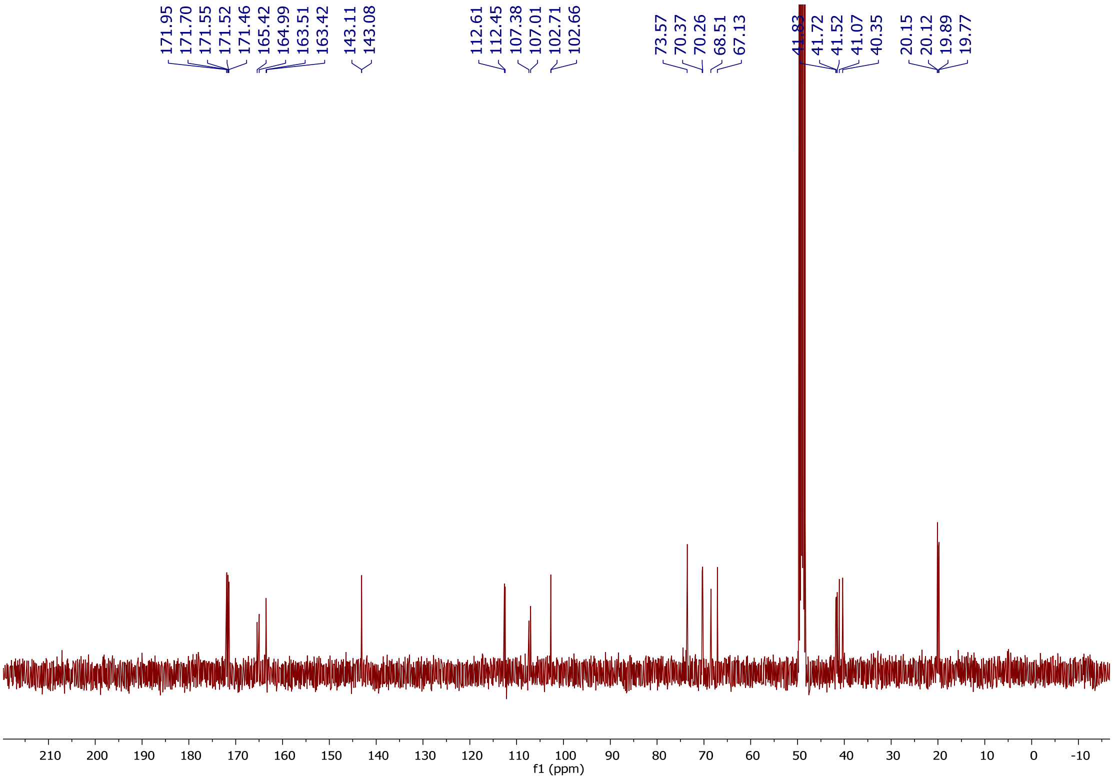

Figure S28. ^{13}C NMR spectrum of compound **8**.

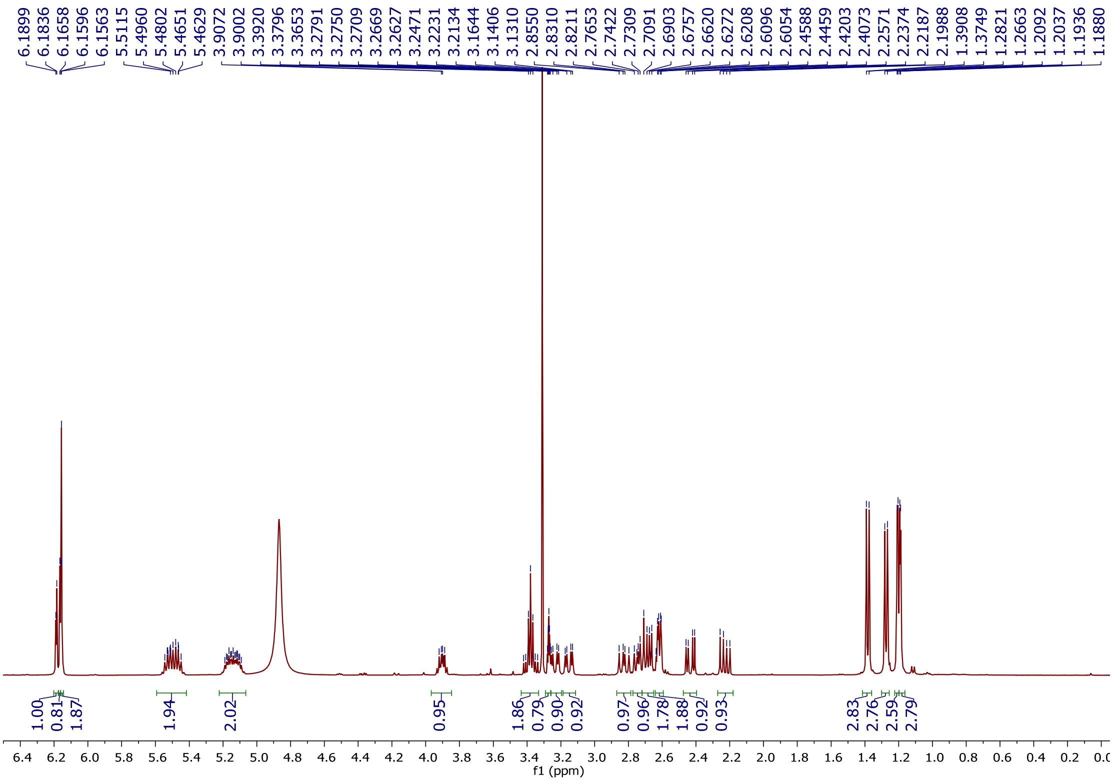

Figure S29. ^{1}H NMR spectrum of compound **9**.

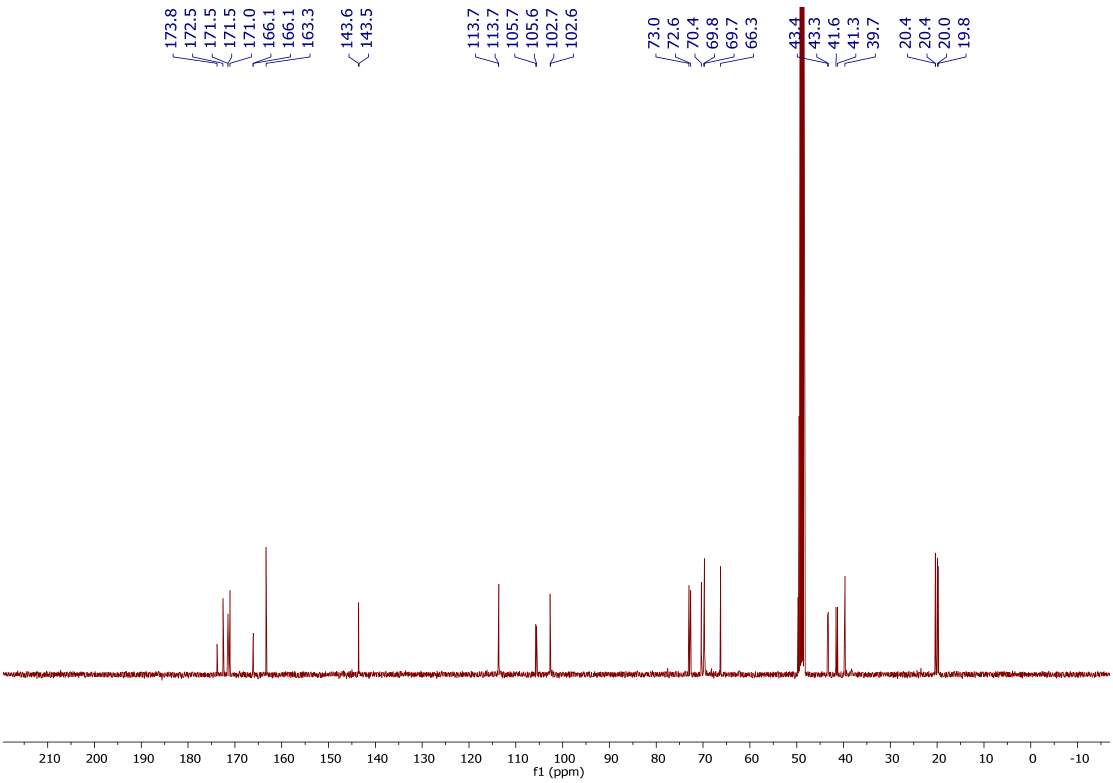

Figure S30. ^{13}C NMR spectrum of compound **9**.

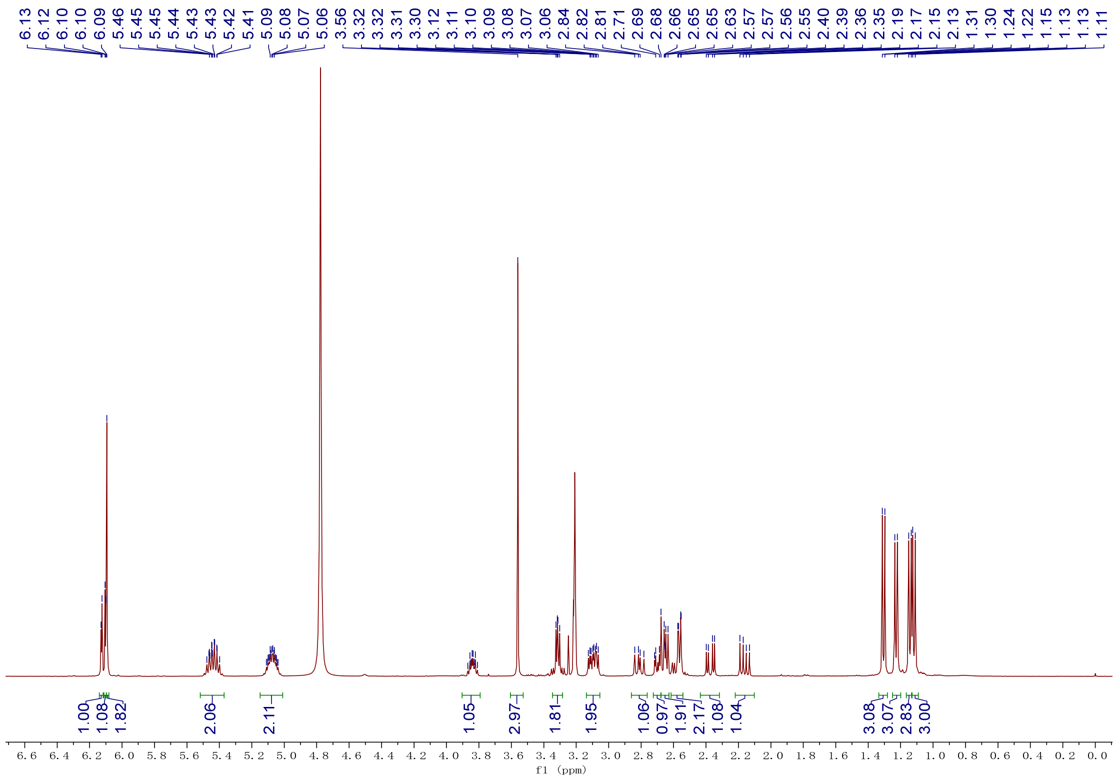

Figure S31. ^{1}H NMR spectrum of compound **10**.

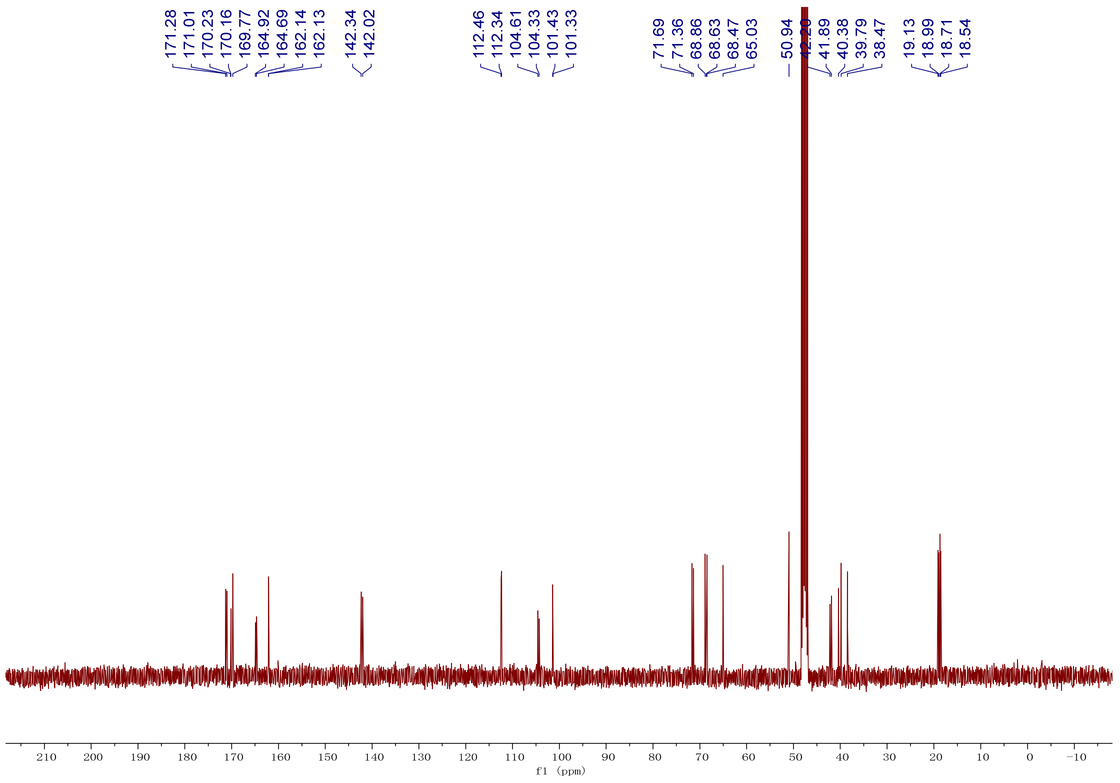

Figure S32. ^{13}C NMR spectrum of compound **10**.

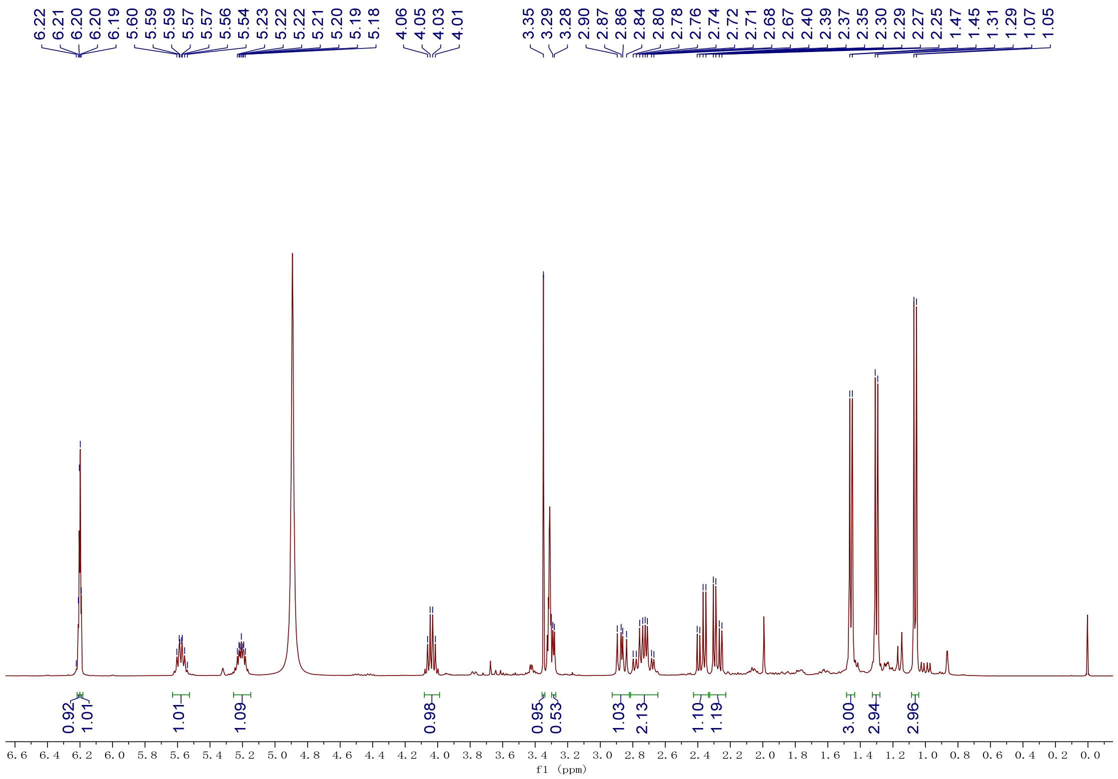

Figure S33. ^{1}H NMR spectrum of compound **11**.

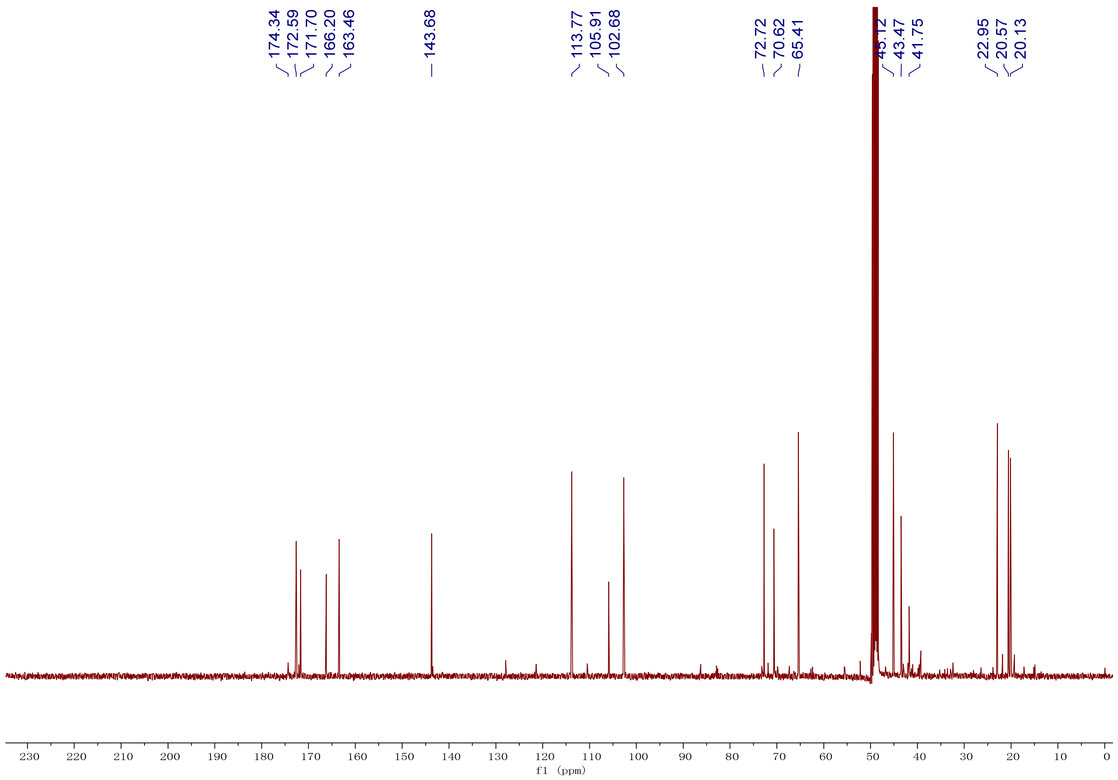

Figure S34. ^{13}C NMR spectrum of compound **11**.

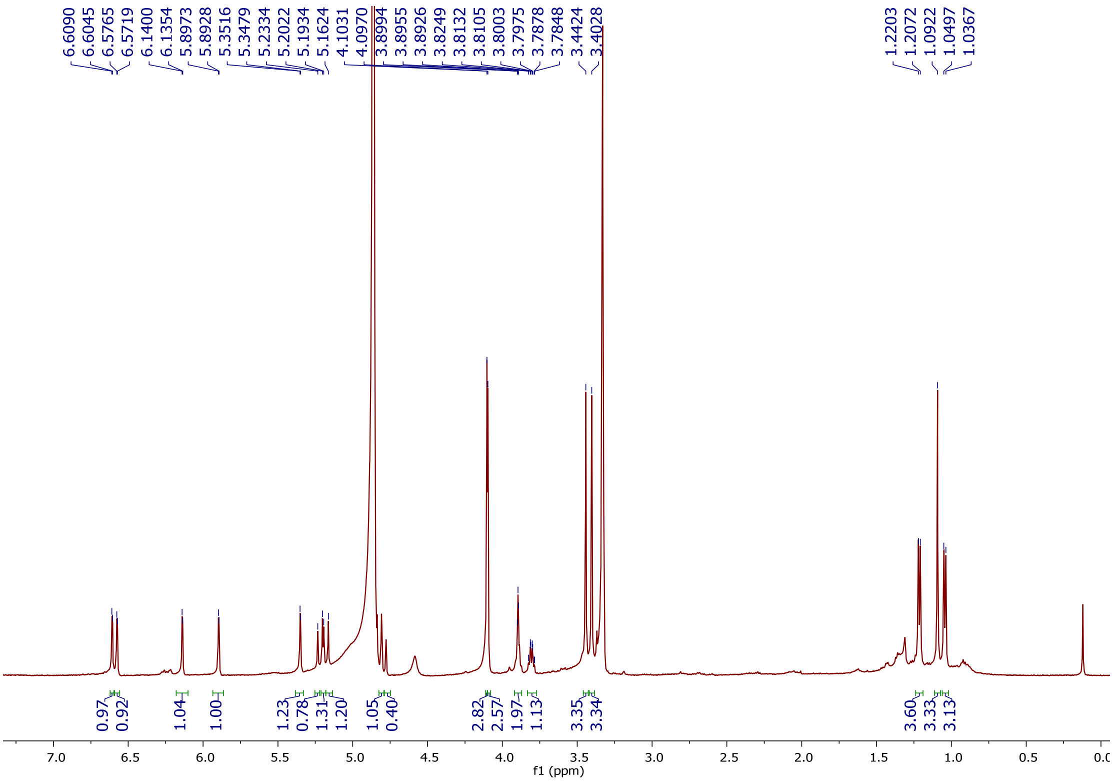

Figure S35. ^{1}H NMR spectrum of compound **12**.

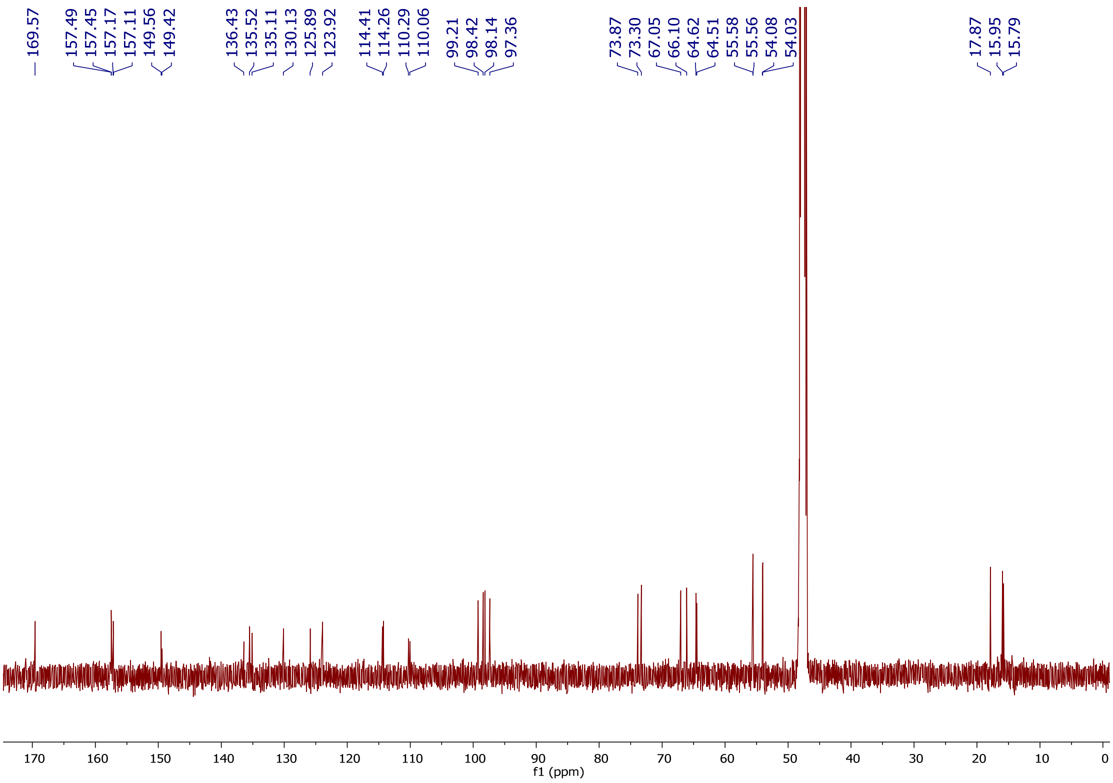

Figure S36. ^{13}C NMR spectrum of compound **12**.

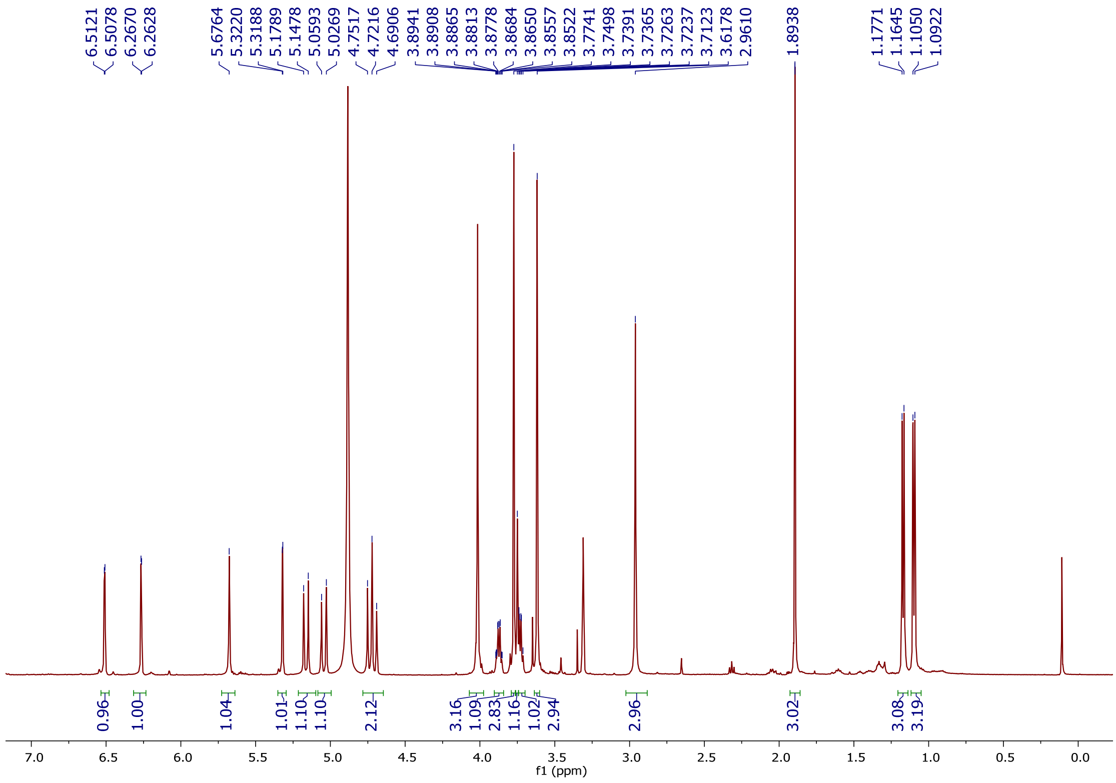

Figure S37. ^{1}H NMR spectrum of compound **13**.

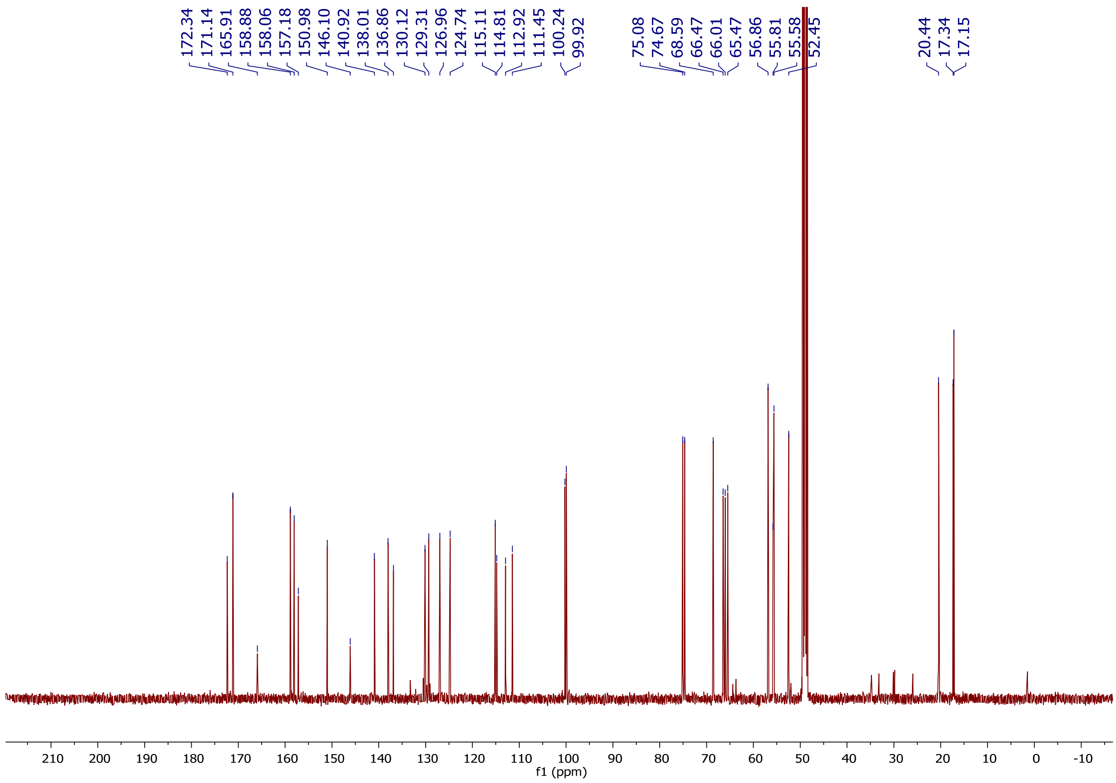

Figure S38. ^{13}C NMR spectrum of compound **13**.

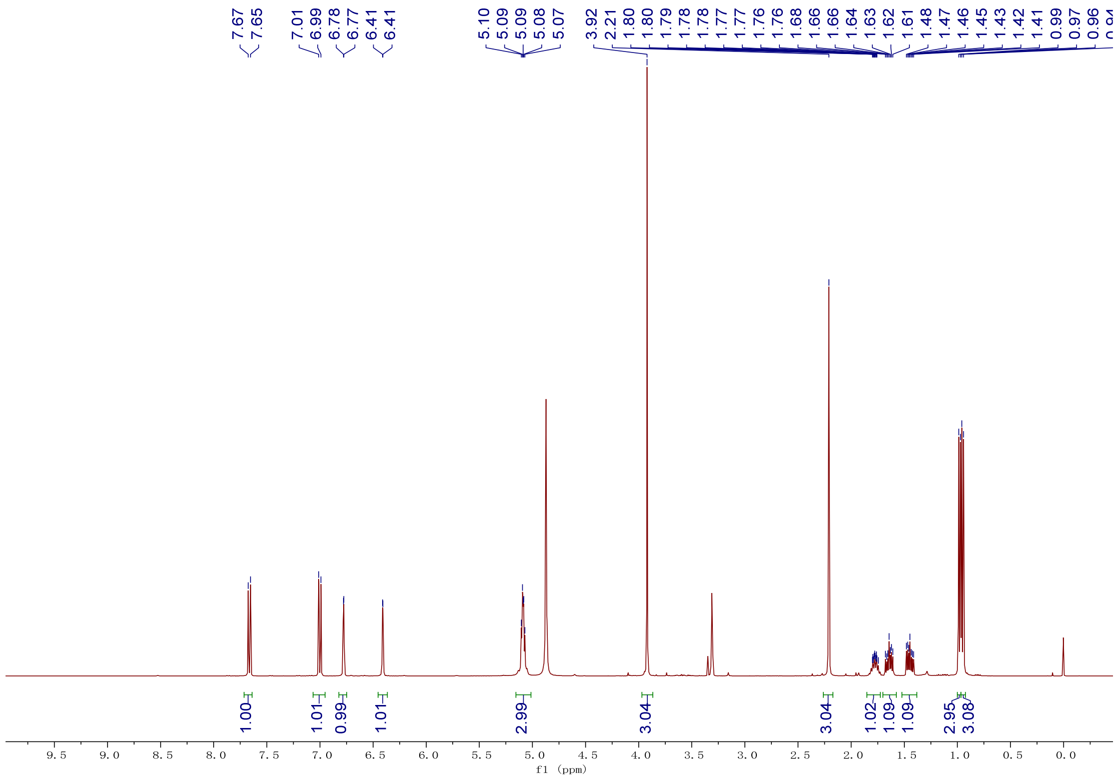

Figure S39. ^{1}H NMR spectrum of compound **14**.

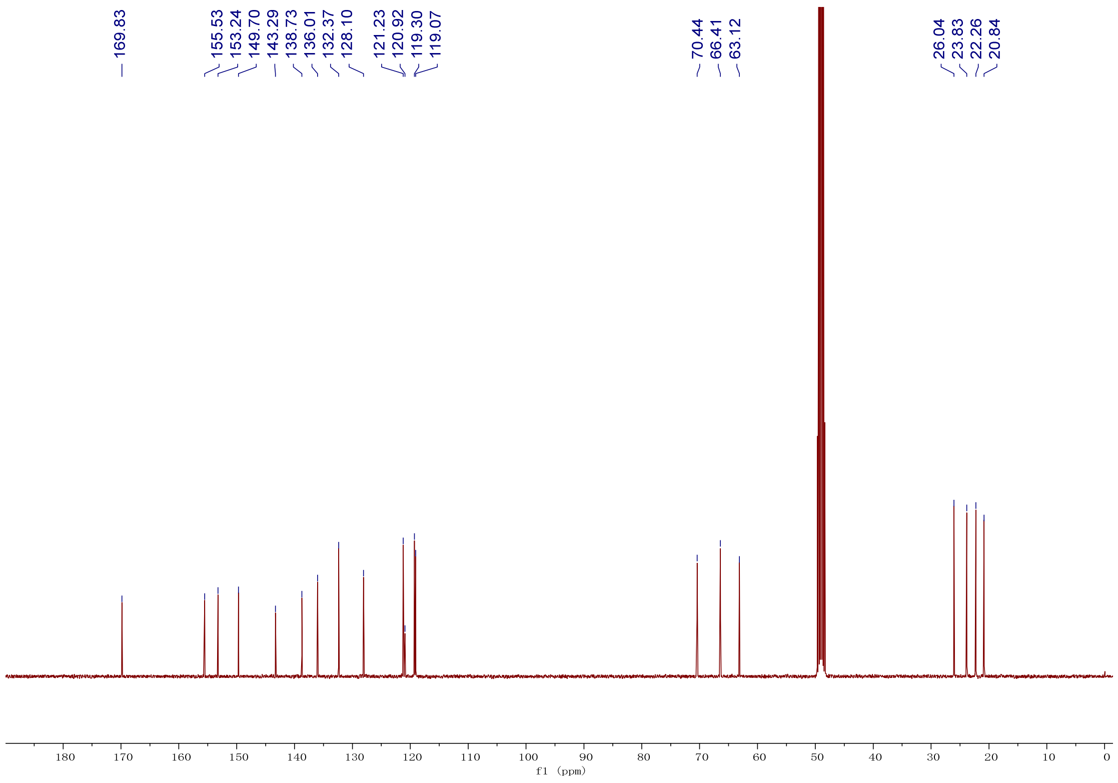

Figure S40. ^{13}C NMR spectrum of compound **14**.

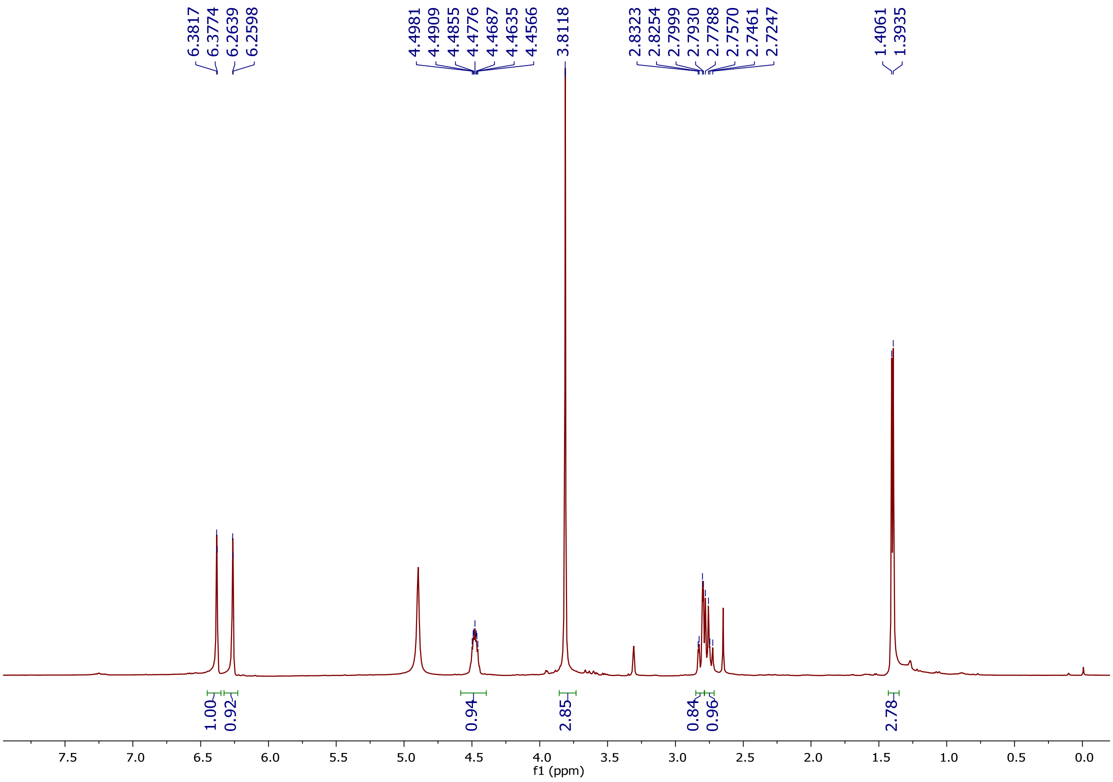

Figure S41. ^{1}H NMR spectrum of compound **15**.

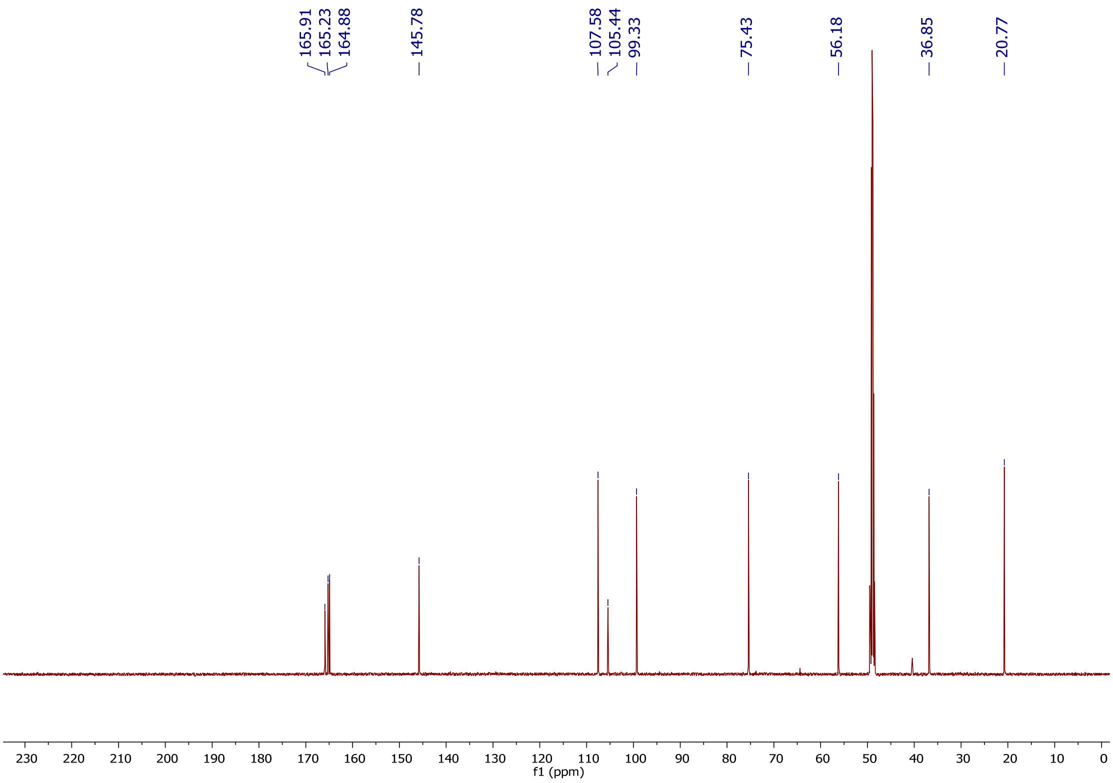

Figure S42. ^{13}C NMR spectrum of compound **15**.

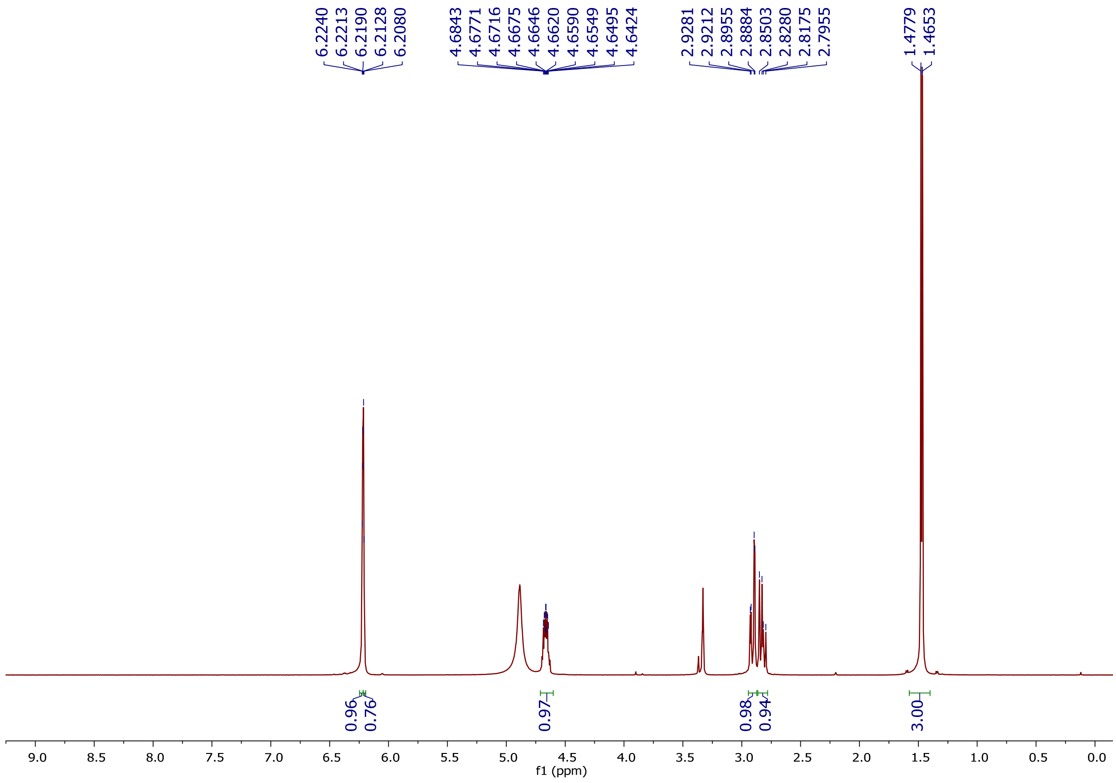

Figure S43. ^{1}H NMR spectrum of compound **16**.

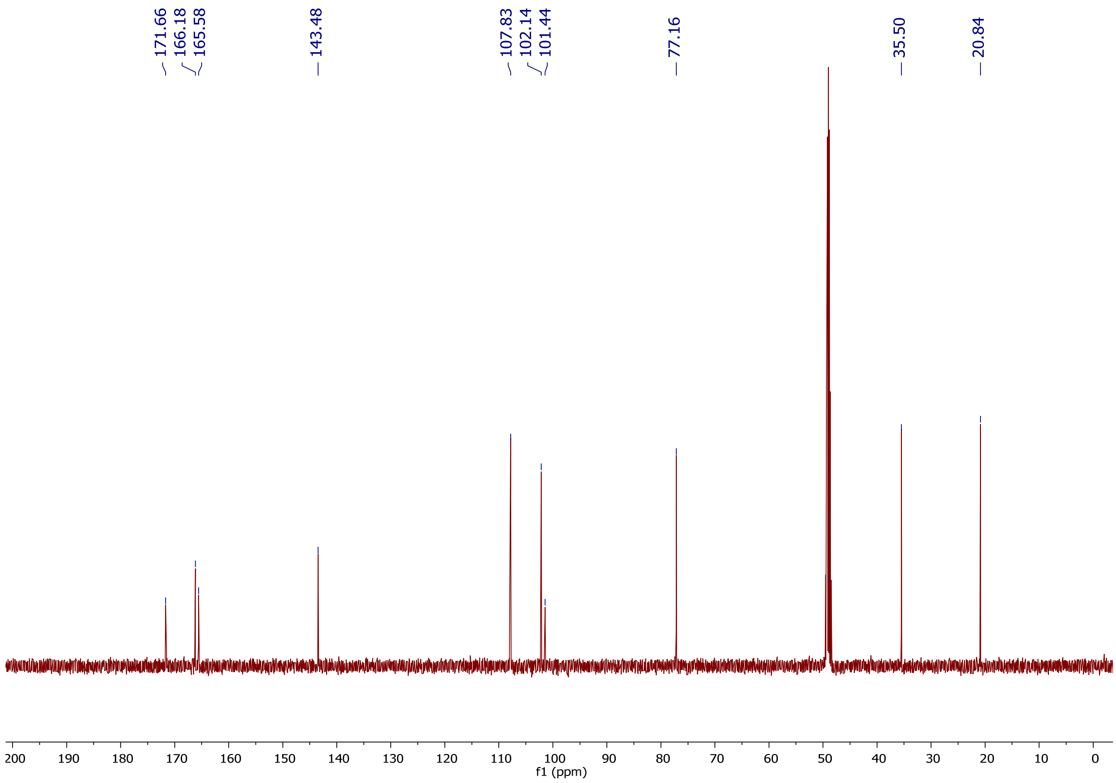

Figure S44. ^{13}C NMR spectrum of compound **16**.
